# Spliced genes in muscle from Nelore Cattle and their association with carcass and meat quality

**DOI:** 10.1038/s41598-020-71783-4

**Published:** 2020-09-07

**Authors:** Danielly B. S. Silva, Larissa F. S. Fonseca, Daniel G. Pinheiro, Ana F. B. Magalhães, Maria M. M. Muniz, Jesus A. Ferro, Fernando Baldi, Luis A. L. Chardulo, Robert D. Schnabel, Jeremy F. Taylor, Lucia G. Albuquerque

**Affiliations:** 1 School of Agricultural and Veterinarian Sciences, São Paulo State University (UNESP), Jaboticabal, SP Brazil; 2 National Council for Scientific and Technological Development (CNPq), Brasilia, DF Brazil; 3 School of Veterinary and Animal Science, São Paulo State University (UNESP), Botucatu, SP Brazil; 4 Division of Animal Sciences, University of Missouri Columbia, Columbia, MO USA

**Keywords:** Animal breeding, Gene expression, Transcriptomics

## Abstract

Transcript data obtained by RNA-Seq were used to identify differentially expressed alternatively spliced genes in ribeye muscle tissue between Nelore cattle that differed in their ribeye area (REA) or intramuscular fat content (IF). A total of 166 alternatively spliced transcripts from 125 genes were significantly differentially expressed in ribeye muscle between the highest and lowest REA groups (p ≤ 0.05). For animals selected on their IF content, 269 alternatively spliced transcripts from 219 genes were differentially expressed in ribeye muscle between the highest and lowest IF animals. Cassette exons and alternative 3′ splice sites were the most frequently found alternatively spliced transcripts for REA and IF content. For both traits, some differentially expressed alternatively spliced transcripts belonged to myosin and myotilin gene families. The hub transcripts were identified for REA (*LRRFIP1, RCAN1* and *RHOBTB1*) and IF (*TRIP12*, *HSPE1* and *MAP2K6*) have an important role to play in muscle cell degradation, development and motility. In general, transcripts were found for both traits with biological process GO terms that were involved in pathways related to protein ubiquitination, muscle differentiation, lipids and hormonal systems. Our results reinforce the biological importance of these known processes but also reveal new insights into the complexity of the whole cell muscle mRNA of Nelore cattle.

## Introduction

The alternative processing of messenger RNAs (mRNAs) produces several transcripts and protein isoforms that have similar or quite different roles in mammalian tissues^1,2^. RNA sequencing (RNA-Seq), is an accurate method for measuring gene expression and can measure all genes in the transcriptome at the same time. This technology could be used to screen the expression of functional candidate genes and identifying essential molecular processes creating a diversity in pathways leading to different tissue phenotypes. One of the usages of RNA-Seq is the identification of alternative splicing events^3,4^.

The spliceosomal mechanism may produce several isoforms by interpreting the limits between exon and intron in many different ways. For the most part, these distinct interpretations, fall into subgroups of alternative splicing events^5^ which may be classified into: cassette, mutually exclusive and coordinate cassette exons; alternative 5′ and 3′ splice sites; intron retention; or alternative first and last exon events^6,7^. The most common alternative splicing events in mammals are cassette exon and 3′ or 5′ alternative splice sites. Intron retention is a rare alternative splicing event in mammals^5,8^.

Whole transcriptomics analysis through splicing-sensitive identified from RNA-Seq or microarrays dataset, provides the capacity to gain an accurate and deep understanding of the contribution of distinct alternative splicing mechanisms to the developmental specific gene expression programs or establishment of tissue^5^. Several studies have reported the frequencies of different types of alternative splicing events in different species^2,4,7,9,10^. In bovine, alternative splicing events have been associated with variation in mastitis susceptibility^2^, embryonic development^11^ and muscle development^12^. Currently, knowledge concerning the molecular mechanisms regulated by alternative splicing events, especially in the context of individual differences in carcass and meat traits in bovine, is limited.

All along the growth process, the animal's weight and body size increase, and the proportion of tissue, mainly the muscle tissue proportion, changes. Ribeye muscle area (REA) in conjunction with carcass weight provides an indication of carcass muscularity and meat yield^13^. Identifying and selecting animals with REA genetic advantages could improve carcass quality, thereby increasing the production of high commercial value meat cut. One of the meat quality traits is the intramuscular fat (IF) content, it is representing the sum of triglycerides and phospholipids; it is, the amount of fat stored between inside muscle cells or muscle fibers. The extent of IF content of the ribeye muscle is a primary determinant of meat palatability and consumer satisfaction with the eating experience. Muscle lipid traits, which determine meat flavor and contribute to beef color, can influence the juiciness and tenderness of the meat^14^.

REA development and IF content are polygenic traits controlled by multiple genes directly (or not) related in muscle and fat biological. However, some genes and biological process that have yet to be elucidated, particularly alternatively spliced genes that are cell-tissue specific. It could play an essential role in overall biological systems. Most of the Brazilian herd is a composite of Zebu breeds, notably among them, the Nelore breed that has been shown to be efficient in terms of rusticity and adaptability to the Brazilian pasture conditions, in addition to presenting excellent maternal ability. The principal bovine breed in Brazil is Nelore cattle, because of its strategic importance to Brazil's GDP (Gross Domestic Product) as the one of the world’s biggest exporter of beef. Nelore cattle became more commercially competitive, then cattle ranchers and researchers have been looking for novel strategies to increase and improvement production, as example, the improvement of different carcass and meat traits.

Zebu animals, as Nelore breed, show a higher frequency of genes/alleles that are unfavorable to carcass and beef quality traits, such as REA development and IF content, when compared to Taurine breeds. Hence, understanding the functional and biological process from spliced genes that may control REA development and IF content in Nelore cattle is a compelling issue in meat science because this knowledge is not yet clear. Wherefore, we used RNA-Seq approach to find candidate genes that produced alternatively spliced transcripts that were differentially expressed in the Nelore’s ribeye muscle that differed for REA and IF content.

## Results

### RNA-seq quality control

Table 1 shows the descriptive statistics for the mean alignment rates for the samples by trait (REA and IF), for details, see Supplementary Figures S1 and S2. In summary, after filtering, the mean number of paired-end reads each sample was approximately 25 million reads. 88% of the trimmed reads, it was uniquely mapped to either the bovine reference genome (UMD3.1.1 + Chr Y). The overall alignment rate was 96.5% (approximately 21.6 million reads mapped in pairs).

**Table 1:** Descriptive statistics for the overall alignment percentage for RNA-Seq data from Nelore cattle samples.

Animals group	Mean of raw reads pairs (Mb)	Mean trimmed reads pairs (Mb)	Overall alignment rate (%)	Mean sample Coverage (X)	Mean mapping quality (Phred score)	Uniquely mapped (%)
REA (N = 30)	25,798,305	22,468,708	96.5	63.85	58.7	88
IF (N = 30)	24,911,474	21,976,535	96.4	65.42		

The coverage refers to the transcriptomics. Phred score = − 10 × Log10.

*REA* Ribeye muscle area, *IF* intramuscular fat content, *Mb* megabase pair, *Mean sample Coverage* mean of number of unique reads that include a given nucleotide in the reconstructed sequence.

The details about quality control indicators and bias estimations (transcript coverage, junction analysis, reads genome origin, and 5′–3′ bias computation) which are specific for RNA-Seq data were summarized in Supplementary Figures S3, S4 and S5. In accordance with Qualimap2^15^ reports, on overage, 65% and 22% of reads were mapped within the exonic and intronic regions of the genes, respectively. Another 13% of reads were mapped to intergenic regions. Mean of number of reads with splice junctions was approximately 16.8 million reads. Splicing junction analysis showed, approximately, 24% novel, 66% known and 10% partly known splicing junctions (for details, see Supplementary Figures S3 and S4).

### Ribeye area (REA)

**Figure 1:**
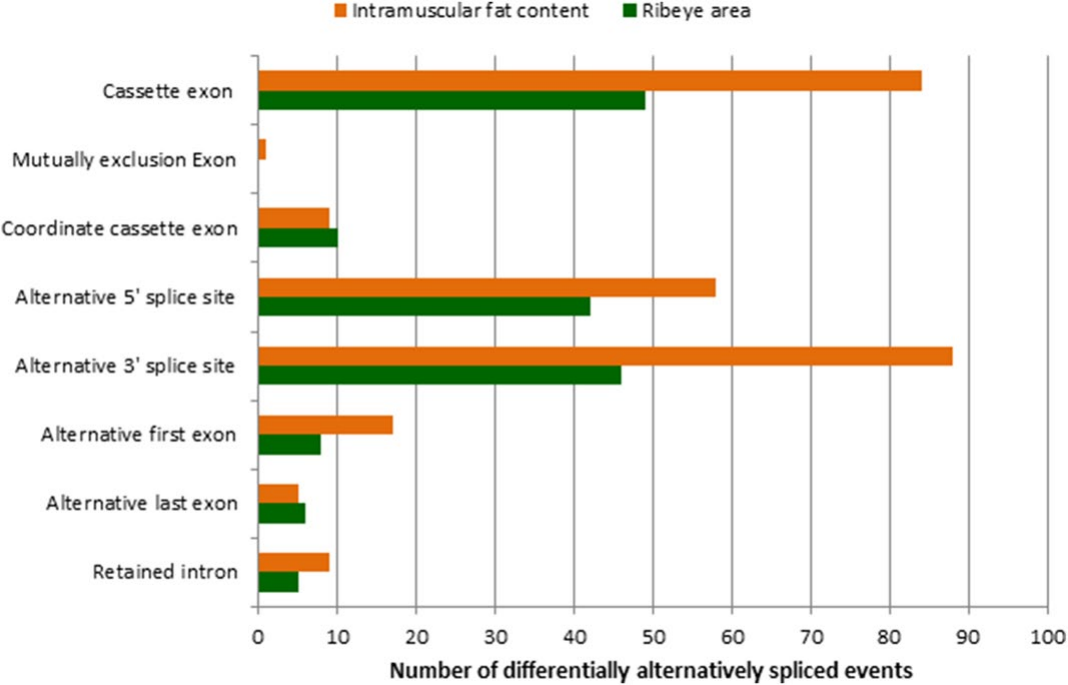
Differentially expressed alternatively spliced transcripts identified in the ribeye muscle of Nelore bulls with the highest and lowest ribeye area (green); or highest and lowest intramuscular fat content (orange). p < 0.05, t-test.

There were 166 alternatively spliced transcripts produced from 125 genes that were differentially expressed between the high ribeye area (HREA) and low ribeye muscle area (LREA) groups (the comprehensive list of the differentially expressed alternatively spliced transcripts associated with REA is provided in Supplementary Table S1). For REA, cassette exon was the most frequently found alternative splicing event (Fig. 1). Of the differentially expressed alternatively spliced transcripts (DAS), 54 were known and 112 were novel transcripts.

Some DAS transcripts belong to important gene families, such as myosin (*MYBPC1* and *MYH1*), myotilin (*MYOT*) and myomesin (*MYOM2*) have an important role in the stability of thin filaments along muscle contraction. The transcript isoforms produced by these gene families differ by cassette exon, intron retention, alternative 3′ and 5′ splice sites events (see Supplementary Table S1). The solute carrier (SLC) gene family (*SLC25A2, SLC25A25, SLC29A1* and *SLC29A2*) produced DAS transcripts with alternative 3′ splice sites. Solute carrier gene family play roles as transporters^16^. Among DAS transcripts, we found *BOLA-DOA* (alternative 3′ splice site). In cattle, *BOLA* is a major histocompatibility complex class II gene that exhibits very little sequence variation, especially at the protein level^17^.

For bulls selected on REA, the biological process Gene Ontology (GO) terms for the DAS genes identified in the pairwise comparison is summarized in Supplementary Table S2. We observed GO terms and pathways related to muscle growth and muscle protein ubiquitination, for example: regulation of protein stability (GO:0031647), smooth muscle tissue development (GO:0048745), protein ubiquitination involved in ubiquitin-dependent protein catabolic process (GO:0042787), cAMP (3′,5′-cyclic adenosine monophosphate) signaling (bta04024), focal adhesion (bta04510) and ECM-receptor (extracellular matrix-receptor) interaction (bta04512), among others.

The analysis also found biological GO terms and pathways related to complex immunological processes, neurological and hormonal systems, such as: adrenergic signaling in cardiomyocytes (bta04261); oxytocin signaling (bta04921) and inflammatory mediator regulation of TRP channels (bta04750). Calcium/Calmodulin Dependent Protein Kinase II Beta (*CAMK2B*)—represented by a novel cassette exon transcript isoform—and Adenylate Cyclase 2 (*ADCY2*)—represented by novel alternative 3′ splice site isoform—were present in the pathways mentioned above and also in six other pathways (Supplementary Table S2).

**Figure 2:**
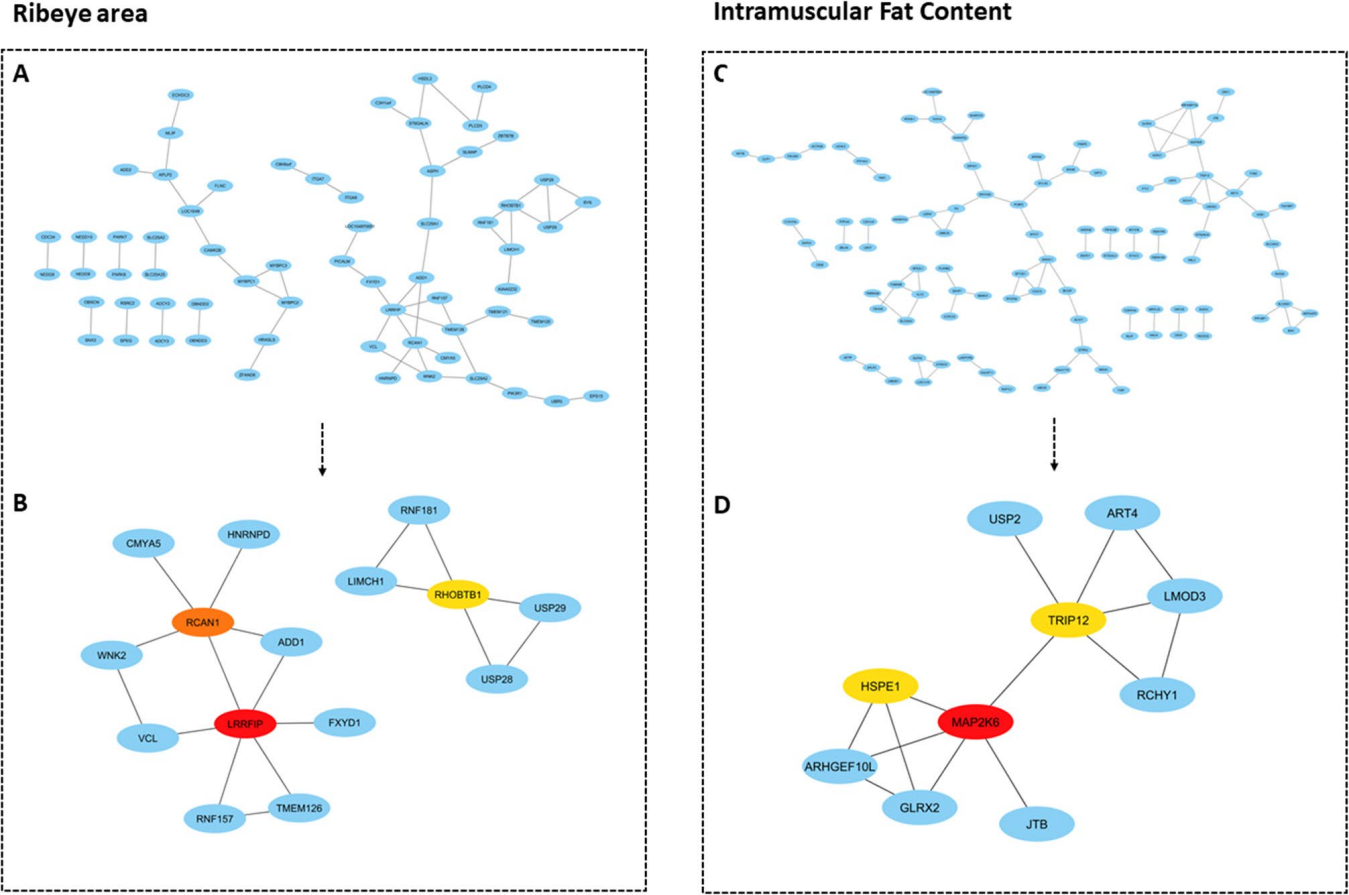
Alternatively spliced genes co-expression networks. (**A**,**C**) The nodes represent the differentially expressed alternatively spliced genes and the lines represent the interaction between nodes. (**B**,**D**) Genes with the most interactions in the network (highest degree), were identified as hub genes for each trait (ribeye area and intramuscular fat content). The top three hub genes were chosen to define the subnetwork (top modules), as this represents the most functional elements of the network for each trait. The more forward ranking is represented by a redder color.

**Figure 3:**
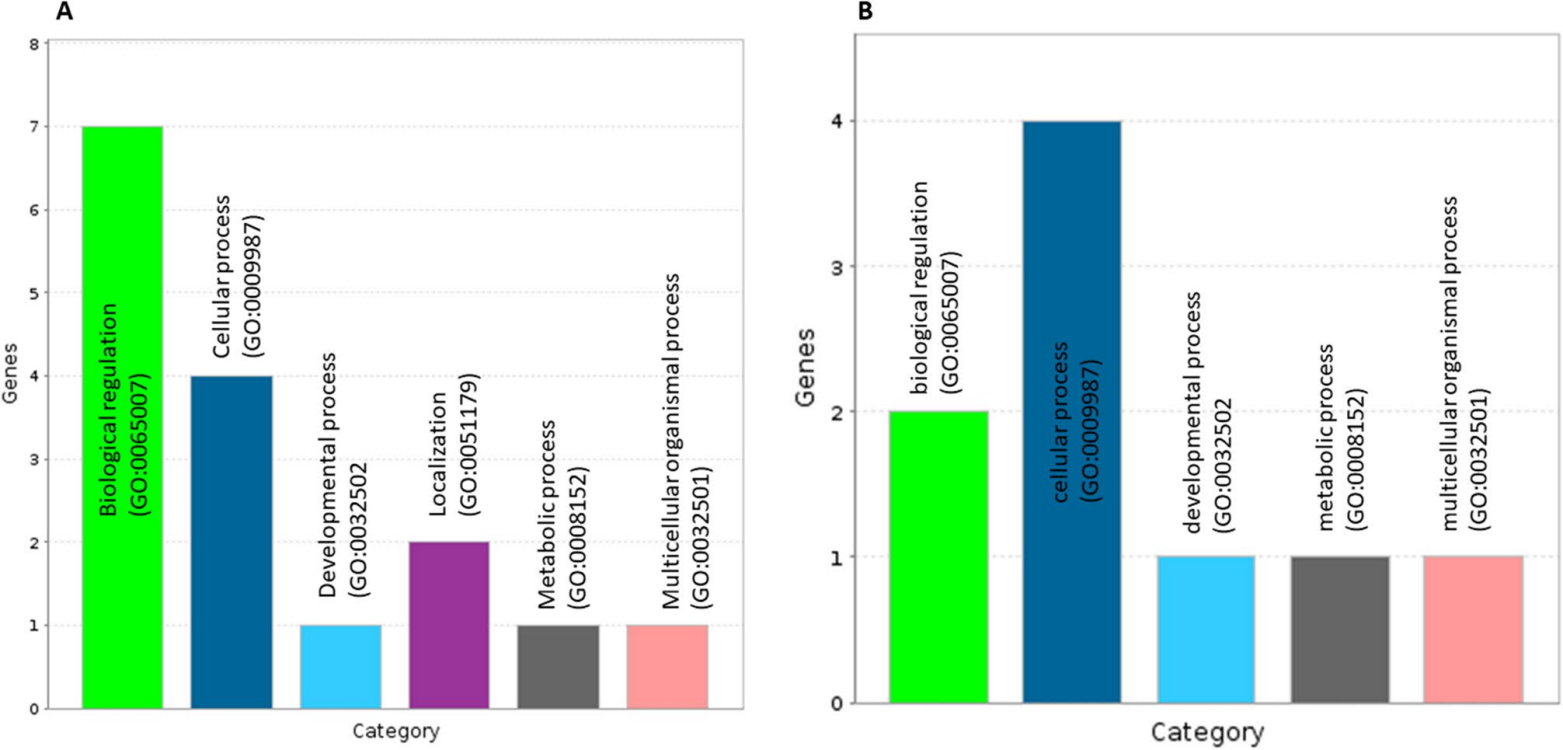
Biological process from alternatively spliced genes present in subnetwork. (**A**) Ribeye area. (**B**) Intramuscular fat content.

Co-expression network analyses found 61 significantly correlated DAS genes pairs (edges) involving 65 genes (nodes) for REA, density = 0.030 and clustering coefficient = 0.130 for REA (Fig. 2A). Networks with clustering and density values close to 1 may contain many edges and nodes connected^18^. Clustering and density values of the network were close to zero, it means that some genes (nodes) are isolated, that is, at that moment they are not being co-expressed with the set of genes. From co-expression network the hub DAS genes were predicted and then, the top three hub DAS genes were chosen to define the subnetwork^18^ (Fig. 2B). The subnetwork was mapped onto biological processes, which had clear associated: biological regulation (GO:0065007), among others (Fig. 3A).

We predicted three top hub DAS genes: LRR Binding FLII Interacting Protein 1 (*LRRFIP1*), Regulator of Calcineurin 1 (*RCAN1*) and Rho Related BTB Domain Containing 1 (*RHOBTB1*). The high interconnection between hub genes and nodes within network, have been shown to be biologically significant^19^. The hub DAS genes identified, could have putative functions as regulators on genes that play a role in development and growth of muscle cells. In general, *LRRFIP1, RCAN1* and *RHOBTB1* have functions in the proliferation of smooth muscle cells^20^, and interact with the calcineurin A^21^ and actin filament systems^22^, respectively.

**Figure 4:**
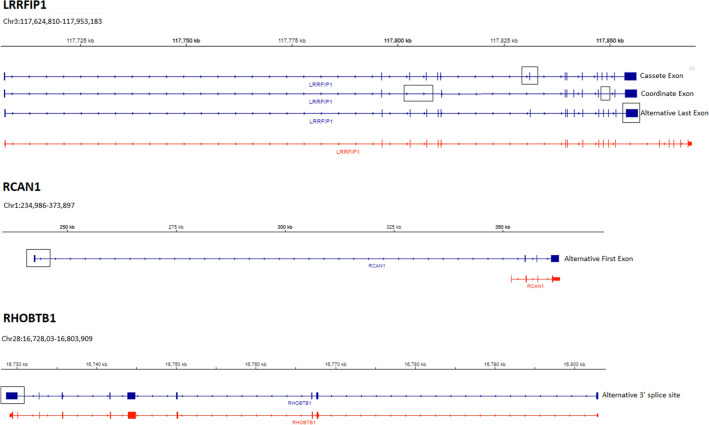
Differentially expressed alternatively spliced transcripts for hub genes associated with ribeye area in Nelore bulls (From Integrative Genomics Viewer (IGV), edited). Blue strand are alternatively spliced transcripts found this study. Red strand is RefSeqGenes (UMD3.1.1 *Bos taurus*). *LRRFIP1* (+ strand): known cassette exon, coordinate cassette exon and alternative last exon. *RCAN1* (+ strand): known alternative first exon. *RHOBTB1* (− strand): novel alternative 3′ splice site. All alternatively spliced exons were highlighted in black square or rectangle.

Figure 4 showed the different DAS transcripts for hub genes associated with ribeye area in Nelore bulls. For the *LRRFIP1* three known DAS transcripts: alternative last exon, cassette exon and coordinate cassette exon. Cassette exon transcripts were more abundant in the HREA animals (see Supplementary Table S1), whereas alternative last exon and coordinate cassette exon transcripts were more abundant in the LREA animals. In accordance with annotation produced by the Ensembl, ten different transcript variants were produced by *LRRFIP1*, previously, in cattle.

*RCAN1* has two known transcript variants annotated in cattle, we found one *RCAN1* produce transcript that differ by known alternative first exons, which were more abundant in the LREA animals (see Supplementary Table S1). Eight transcript isoforms were produced by *RHOBTB1* in accordance with annotation produced by the Ensembl. This study identifies a novel alternative 3′ splice sites for this gene, predicted a putative novel transcript. The alternative 3′ splice sites usage in *RHOBTB1* was more abundant in the LREA animals (see Supplementary Table S1).

### Intramuscular fat content (IF)

A total of 269 (78 known and 191 novel) alternatively spliced transcripts from 219 genes were differentially expressed between highest intramuscular fat (HIF) and lowest intramuscular fat (LIF) animals (see Supplementary Table S3). Alternative 3′ splice site was the most frequently observed alternative splicing event found in the IF content analysis (Fig. 1).

Members of myosin (*MYH7B, MYLK2* and *MYO18A*) and myotilin (*MYOT*) gene families were among the DAS transcripts. These genes, produced transcripts that differed by alternative first exons, alternative 3′ splice sites, alternative 5′ splice sites and retained introns. In addition to these, members of the myozenin (*MYOZ1* and *MYOZ3*) family were found to produce DAS transcripts: alternative first exons, alternative 3′ and 5′ splice sites. These genes play an important role in the modulation of calcineurin signaling. Another DAS transcripts belong ubiquitin family (*UBAC2* and *UBE3C*). These genes produced transcripts with cassette exons, alternative 3′ splice sites and alternative 5′ splice sites. The ubiquitin–proteasome system plays an important role in muscle loss^23^.

Most biological terms found for animals selected to differ for IF content were associated with growth, regulation and proteolysis of muscle cells (Supplementary Table S4), for example: myofibril assembly (GO:0030239); skeletal muscle cell differentiation (GO:0035914) and ubiquitin-dependent protein catabolic process (GO:0006511). The analysis found GO terms and pathways related to lipid metabolism, such as PI3K-Akt (bta04151), mechanistic target of rapamycin (mTOR) signaling (bta04150) and negative regulation of TORC1 signaling (GO:1904262). Serine/Threonine Kinase 11 (*STK11*) and Eukaryotic Translation Initiation Factor 4B (*EIF4B*) produce transcript isoforms with alternative 3′ splice sites and were present in these pathways. All of the GO terms and pathways mentioned above play important roles in muscle and lipid metabolism.

For IF, there were 92 significantly correlated gene pairs (edges) involving 101 genes (nodes), density = 0.018 and a clustering coefficient = 0.152 (Fig. 2C). Clustering and density values of the network were close to zero, it means that some nodes are isolated, that is, at that moment they are not being co-expressed with the set of genes^18^. Hub DAS genes within the co-expression network were predicted. From the first three hub DAS genes were chosen to define the subnetwork^18^ (Fig. 2D). The three hub DAS genes were: Thyroid Hormone Receptor Interactor 12 (*TRIP12*), Heat Shock Protein Family E member 1 (*HSPE1*) and Mitogen-Activated Protein Kinase Kinase 6 (*MAP2K6*). *TRIP12*, *HSPE1* and *MAP2K6* genes are involved in ubiquitin mediated proteolysis^24^, facilitates the correct folding of imported proteins^25^, the regulation of the mitogen-activated protein kinase pathway^26^, respectively. The subnetwork genes (Fig. 2D) were mapped onto biological processes, which include biological regulation (GO:0065007) and cellular processes (GO:0009987), among others (Fig. 3B).

**Figure 5:**
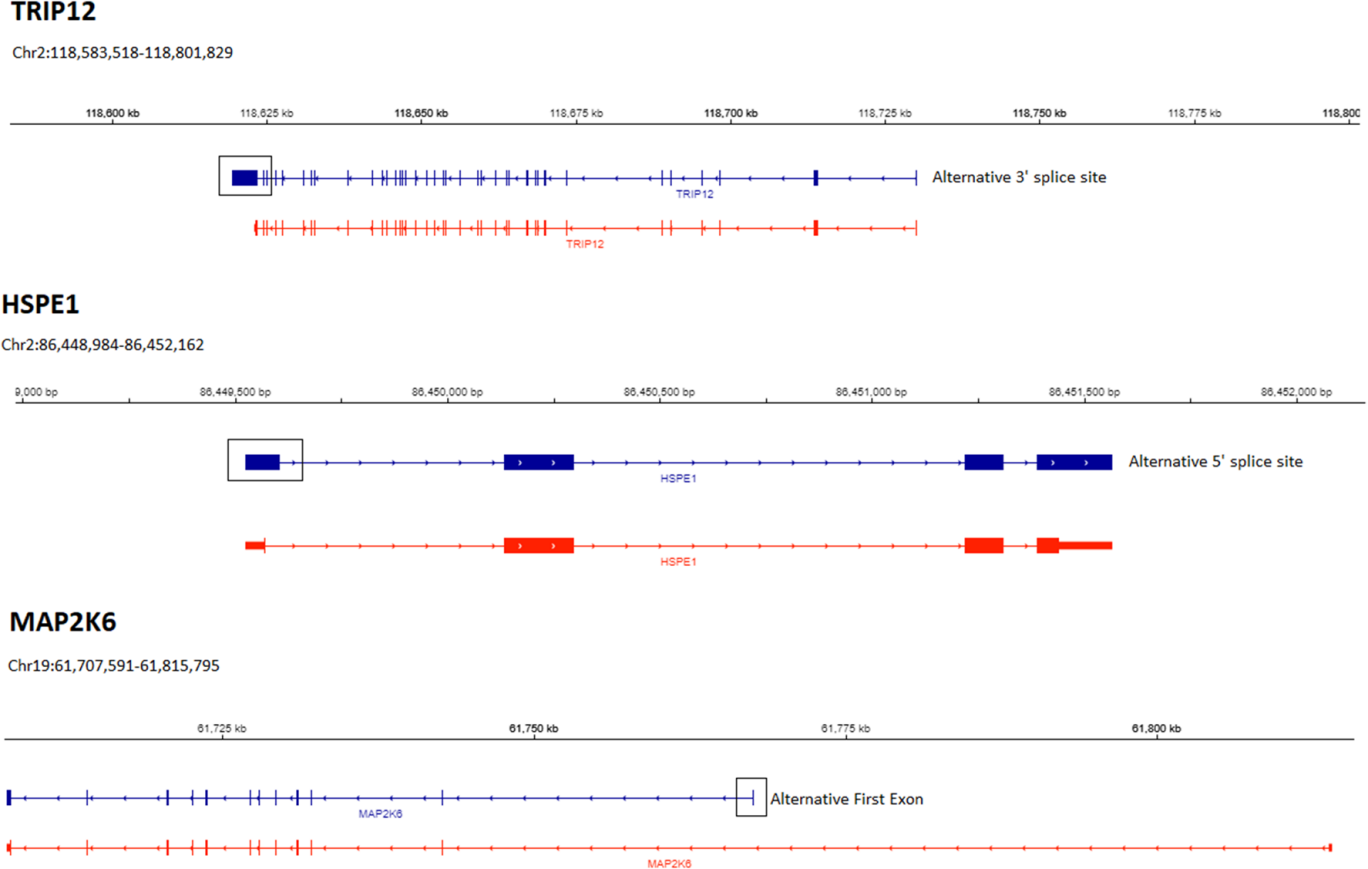
Differentially expressed alternatively spliced transcripts for hub genes associated with intramuscular fat content in Nelore bulls [From Integrative Genomics Viewer (IGV), edited]. Blue strand are alternatively spliced transcripts found this study. Red strand is RefSeqGenes (UMD3.1.1 *Bos taurus*). *TRIP12* (− strand): known alternative 3′ splice site. *HSPE1* (+ strand): novel alternative 5′ splice sites. *MAP2K6* (− strand): known alternative first exon. All alternatively spliced exons were highlighted in black square or rectangle.

The Fig. 5 showed the different DAS transcripts for hub genes associated with IF content. For the *TRIP12* one known DAS transcript that differed by their use of alternative 3′ splice sites. This transcript was more abundant in the HIF animals (see Supplementary Table S3). For *HSPE1* a novel DAS transcript: alternative 5′ splice. In accordance with annotation produced by the Ensembl, three different transcript variants were produced by *MAP2K6*, previously, in cattle. In this study, one of these transcripts was differentially expressed: alternative first exons, being more abundant in the LIF animals (see Supplementary Table S3).

## Discussion

Differentially expressed alternatively spliced transcripts and their associated genes were identified in this study, using RNA-Seq data from the ribeye muscle of Nelore bulls phenotypically divergent for REA or IF content. In cattle, most genes have multiple isoforms characterized by different event types. The cassette exon and alternative 3′ splice site events were frequently responsible for the transcript isoforms found to be differentially expressed in the ribeye muscle of bulls with the highest and lowest REA or IF content.

Cassette exon and alternative 3′ splice site are the most common alternative splicing events in mammalian pre-mRNAs^8^. This case, the exons are flanked on two side (constitutive and competing alternative splice sites). The exons are included or excluded from the mature RNA leading to extended or shortened transcripts variants. Bland et al.^27^ studied mice myogenic differentiation and found 95 alternative splicing events. Most of the splicing events found for Bland et al.^27^ was cassette exons (86%).

The co-expression analyses were performed with gene sets that contained the isoforms that were differentially expressed between the highest and lowest REA and IF content groups. Through co-expression analyses hub DAS genes for each trait were predicted that may be the primary regulators of the other genes with differentially expressed transcript isoforms found in this study. Hub genes are expected to play significant functions in organism’s^28^. In addition, a functional enrichment analysis was performed for the gene sets found for each trait. In general, for both traits, the results indicated that hub DAS genes had functions in protein ubiquitination, muscle differentiation and regulation of lipolysis in adipocytes.

This study also found biological terms and pathways related to immune function systems, indicating the pleiotropic nature of the genes and their isoforms in several phenotypes. Several of the genes that were found to produce differentially expressed splice transcripts in this study; have not previously been reported to be associated with carcass or meat traits of beef cattle. For example, *CAMK2B* (differentially expressed in animals phenotypically divergent for REA and represented by novel cassette exon events) was present in nine pathways (Supplementary Table S2) related to the neurological, hormonal, and immune systems, including: adrenergic signaling in cardiomyocytes (bta04261); oxytocin signaling (bta04921) and inflammatory mediator regulation of TRP channels (bta04750).

*CAMK2B* has been associated with neurodevelopmental disorders in humans^29^. *CAMK2B* is a serine/threonine kinase that is highly abundant in brain, especially in the post synaptic density. Different isoforms of this gene have distinct cellular localizations and interact differently with calmodulin^29^. Calmodulin mediates many key processes, such as, metabolism, smooth muscle contraction, apoptosis, short-term memory, long-term memory, intracellular movement and the immune response^30^.

Another gene that has not previously been reported to be associated with carcass or meat traits of beef cattle is Serine/Threonine Kinase 11 (*STK11*). This gene was differentially expressed between animals phenotypically divergent for IF content and by a known isoform with an alternative 3′ splice site, regulates cell polarity and functions as a tumor suppressor^31^. *STK11* is involved in TORC1 signaling (GO:1904262); mTOR signaling (bta04150) and PI3K-Akt signaling (bta04151) (see Supplementary Table S4). All of these processes have important roles in the growth, proliferation, motility and survival of cells.

Several other genes that were predicted to produce alternatively spliced isoforms that were differentially expressed have also been implicated in muscle differentiation and growth; protein ubiquitination in muscle; and lipid metabolism. All of these processes could promote changes in ribeye muscle area and intramuscular fat content phenotypes.

### Ribeye area

Several differentially expressed alternatively spliced transcripts were produced by myosin (*MYBPC1* and *MYH1*), myotilin (*MYOT*), myomesin (*MYOM2*), solute carrier (*SLC25A2, SLC25A25, SLC29A1* and *SLC29A2*) and *BOLA* (*BOLA-DOA*) genes families. Myosin, myotilin and myomesin proteins have a significant role in the stability of thin filaments during muscle contraction and are the main components of myofilaments^32,33^. *SLC* gene family play roles as transporters and have been associated with REA^34^ in Nelore cattle. *BOLA-DOA* belongs to the major histocompatibility complex and has effects in the immune system. Members of the *BOLA* gene family have previously been associated with tenderness^35^ in Nelore cattle and growth traits in Limousin, Simmental Charolais, Holstein, Angus and Hereford, beef cattle^36,37^.

Three hub genes (*LRRFIP1, RCAN1* and *RHOBTB1*) with alternatively spliced transcripts were differentially expressed between Nelore bulls selected to differ for REA. The differences in transcript splicing event usage for *LRRFIP1, RCAN1* and *RHOBTB1* found here may be due to the tuning of the spliceosome machinery. The spliceosome is a complex snRNA. The introns are removed from pre-mRNAs through two consecutive phosphoryl transfer reactions. The spliceosome is assembled de novo on a imature mRNA for each splicing event and a complex series of assembly steps result to the active conformation.

Hub genes *LRRFIP1* and *RHOBTB1* play roles in muscle cell development and regulation in humans and mice^21,23^. *LRRFIP1* performs the function of transcriptional repressor who has binding affinity with GC-rich consensus sequence and can control the proliferation of smooth muscle cells^21^. We found known alternative last, cassette and coordinate cassette exons transcripts produced by *LRRFIP1*. For *LRRFIP1*, alternative last and coordinate cassette exons transcripts were more abundant in the LREA animals, this result showed, these transcripts could be playing a role as repressor of some genes that function in proliferation of smooth muscle cells, whereas, cassette exon transcript was more abundant in HREA animals. This result could be explained by the fact that for some genes, to initiate transcription, the presence of an activator is required. For others, it will be avoiding the connection of a repressor. These cases are referred to as down-regulation because it is the absence of a protein that allows transcription to occur.

The protein encoded by *RHOBTB1* has function in actin filament system organization and small and small GTPase-mediated signal transduction^22^. For this gene, a putative novel transcript (3′ splice sites) was predicted. The alternative 3′ splice sites usage in *RHOBTB1* was more abundant in the LREA animals, which could result in the likely morphological changes that occur in the muscle structure of these animals. *RCAN1* has previously been associated with REA in a Wagyu × Angus F1 population^22^. *RCAN1* produces a protein that inhibits the transcriptional responses of the calcineurin-dependent mediated by the binding to the catalytic domain of calcineurin A.

It is known that calcineurin A signaling is important for the normal function of cardiac and skeletal muscle. In skeletal muscle, calcineurin A is involved in different processes, as dystrophic muscle damage, fiber type specification and myotube differentiation^38^. *RCAN1* has a tissue-specific expression pattern and produces transcripts that differ by known alternative first exons in mice^39^, humans^40^ and cattle^41^. In this study, *RCAN1* transcripts that differ by known alternative first exons were more abundant in the LREA animals, which could inhibit the calcineurin-dependent transcriptional responses, causing muscle loss in this animal group.

The gene set enrichment analysis based on transcript isoforms that were differentially expressed in animals differing for REA revealed GO terms and pathways related to muscle growth and muscle protein ubiquitination (Supplementary Table S2). Skeletal muscle hypertrophy is a process dynamically adapted, which is mainly caused by protein synthesis, which is stimulated by pathways subjected to external factors, growth factors and other anabolic hormones, and neuronal inputs^42^. For example, we found the known and important pathways: focal adhesion (bta04510), cAMP (adenylyl cyclase) signaling (bta04024) and ECM-receptor (extracellular matrix-receptor) interaction (bta04512). The cAMP pathway contributes to an increase in myofiber size and metabolic phenotype over the long term^43^. It also participates in the differentiation of cell muscle precursors^44^. The cellular events described above, are necessary for the efficient regeneration of skeletal muscle^43^.

Focal adhesions, serve as mechanical links to ECM-receptor and other molecules, playing key roles in important biological processes^45^. Focal adhesion kinase is involved in mammalian myoblast fusion in vivo and in vitro^44^. The results show the biological importance of cAMP signaling, focal adhesion and ECM-receptor signaling in determining the composition and organization of bovine muscle tissue.

Pathways related to hormonal systems were also found, among them: oxytocin (bta04921) and glucagon signaling (bta04922). *RCAN1* is a member of the oxytocin signaling pathway is fundamental for the maintenance of homeostasis and regeneration of skeletal muscle tissue in mice^46^. Glucagon signals liver and muscle cells to change stored glycogen into glucose, which is then released into the bloodstream and used by other cells for energy^47^. The protein turnover (synthesis and breakdown) is continuous and ensures the skeletal muscle quality and functional integrity. In addition, the hormones are essential regulators of this remodeling process.

In contrast to muscle growth, muscle loss is an undermining consequence of a range of pathology and metabolic conditions, for example, an increase in protein degradation. We found processes related to the proteolytic system (protein ubiquitination involved in ubiquitin-dependent protein catabolic process—GO:0042787, ubiquitin mediated proteolysis—bta04120 and protein polyubiquitination—GO:0000209), some of which were involved in muscle degradation^48^. Among these, the ubiquitin–proteasome system is very conserved across vertebrates, and works as a degrader of the main proteins of the contractile skeletal muscle, that is, it has an important role in muscle loss^23^.

Genes encoding differentially expressed alternatively spliced transcripts associated with REA were also related to immune function, such as the Adenylate Cyclase 2 (*ADCY2*) gene which also is annotated with several other GO terms and is a member of other pathways including those related to the neural and hormonal systems. There are complex interactions between the skeletal muscle and the immune systems that regulates muscle regeneration^49^. *ADCY2* is a membrane-associated enzyme that catalyzes the formation of cyclic adenosine monophosphate^50^, a secondary messenger. An alternative 3′ splice site event was found for this gene which is a member of the cAMP signaling pathway (bta04024). *ADCY2* may be related in different biological processes, and has previously been associated with meat tenderness in Yanbian cattle^51^.

### Intramuscular fat content

We found differentially expressed alternatively spliced transcripts belonging to members of myosin (*MYH7B, MYLK2* and *MYO18A*), myotilin (*MYOT*), myozenin (*MYOZ1* and *MYOZ3*) and ubiquitin (*UBAC2* and *UBE3C*) families. Skeletal muscle fiber metabolism is a factor that may affect intramuscular fat deposition. There is a positive correlation between the extent of intramuscular fat and oxidative muscle fiber percentage^52^. When the rate of protein deposition decreases (possibly due to ubitiquation processes) lipid deposition becomes the major component of weight gain and the energy request to fat tissues increases^53^.

*TRIP12, HSPE1* and *MAP2K6* which have functions related to cell regulation and maintenance^24–26^ were predicted to be hub genes associated with IF content. *TRIP12* encoded transcript with known alternative 3′ splice site, which was more abundant in the HIF animals. *TRIP12* is a protein coding gene that is a key regulator of the response to DNA damage. The related pathways include Class I MHC mediated antigen processing and presentation, and ubiquitin mediated proteolysis^25^. *TRIP12* transcripts were expressed in the skeletal muscle of Holstein–Friesian, Limousin, Hereford and Polish Red bulls^54^. In humans, *TRIP12* plays a role in the ubiquitin fusion degradation pathway, which is a proteolytic system that is conserved in mammals^55^. Therefore, the *TRIP12* transcripts more expressed in HIF animals could plays role in the immune systems and protein degradation.

This study predicted a putative novel *HSPE1* transcript with alternative 5′ splice site more expressed in HIF animals. SNPs (Single nucleotide polymorphisms) found in *HSP* family genes were associated with thermal tolerance traits in Chinese Holstein^56^ and Angus cattle^57^. Heat shock proteins provide subsidies for cells to identify and promote the refolding of damaged proteins or target them to appropriate proteolytic systems, thereby helping to eliminate proteins whose damage is not suitable for recovery. As long as the animal is under stress, HSP levels will be high. This high expression increases the synthesis and maturation of new proteins that will replace those affected by stress. The increase of HSP in damaged cells not only contributes to protein repair, but also plays an important role in maintaining viability because it inhibits apoptosis^58^. Therefore, the *HSPE1* transcript found in this study could be aiding in the repair of proteins in HIF group.

*MAP2K6* produces three known transcripts one off them differ by known alternative first exons, which were found in this study and more abundant in the LIF animals. Single nucleotide polymorphisms in *MAP2K6* have previously been associated with marbling score, back fat thickness and carcass weight in Hanwoo cattle^59^. *MAP2K6* is a member of the dual specificity protein kinase family and plays a role in the regulation of the mitogen-activated protein kinase pathway^26^. Mitogen-activated protein kinase (MAPK or MAP kinase) is a protein kinase that is specific to the serine and threonine aminoacids. Mapk protein is involved in directing the response of cells to a variety of stimulus, such as osmotic stress, mitogens, proinflammatory cytokines and heat shock. They regulate cell functions including apoptosis, gene expression, proliferation, mitosis, differentiation, and cell survival. Therefore, the *MAP2K6* transcript, as well as the *HSPE1* transcript, found this study, could be aiding in the repair of proteins in LIF animals.

The enrichment analysis suggested that the most relevant function of the genes with significant isoform expression shifts for IF was related to the growth, regulation and proteolysis of muscle cells (Supplementary Table S4), for example: myofibril assembly (GO:0030239); skeletal muscle cell differentiation (GO:0035914) and ubiquitin-dependent protein catabolic process (GO:0006511). This suggests that variation in IF content may be due to a balance between the absorption, synthesis and degradation of intramyocellular and extramyocellular lipids, which involves many metabolic pathways in myofibers^60–62^. Intramuscular fat accumulates both within (intramyocellular) and externally (extramyocellular) of muscle fibers^62^. The intramyocellular lipids are stored as spherical droplets in muscle cells and are reportedly associated with aerobic metabolism, whereas the extramyocellular lipids are located in long fatty septa of laminar shape, along muscle fiber bundles. Intramyocellular lipids play an important role in muscle metabolism^62^.

The PI3K-Akt (bta04151), mTOR signaling (bta04150) and negative regulation of TORC1 signaling (GO:1904262) pathways were overrepresented in this study. *EIF4B* which is a member of these pathways, produces a transcript with a novel alternative 3′-splice site, that was found to be more abundant in the LIF animals. *EIF4B* is a protein coding gene that is required for the binding of mRNA to ribosomes. The activity of EIF4 family genes is controlled by mTOR serine threonine kinase^63^ which forms the mTORC1 and mTORC2 complexes. mTORC1 may play a role in regulating lipid synthesis, which is required for cell growth and proliferation, but this is not completely clear yet^64^. It has been demonstrated that mTORC1 positively regulates the transcription factors (sterol regulatory element binding protein 1—*SREBP1* and peroxisome proliferator-activated receptor-γ—*PPARGγ*) that control the expression of genes encoding proteins involved in lipid and cholesterol homeostasis^65^. The PI3K-AKT pathway is an intracellular signaling pathway essential in regulating the cell cycle. PI3K activation phosphorylates and activates AKT. AKT can have a number of downstream effects such as activating mTOR^66^.

We found candidate genes that encode differentially expressed alternatively spliced transcripts associated with IF content in Nelore cattle. Previous studies have identified differentially expressed genes in Simmental-Luxi cross^67^, Wagyu and Angus^68^, Wagyu and Holstein^69^ and Nelore^70^, suggesting that some of these genes regulate lipid composition and deposition in intramuscular fat^67–70^. These findings and our results enable a better understanding of the mechanisms underlying the gene transcription associated with IF content in Nelore cattle.

## Conclusion

RNA-Seq was used to identify candidate genes that encode alternatively spliced transcripts that were associated with REA and IF content in Nelore bulls. The results suggest that REA and IF content may be influenced by sets of genes that encode alternatively spliced transcripts (both known and novel transcripts). For both traits, we found genes with biological process GO terms that are involved in pathways related to protein ubiquitination, muscle differentiation, regulation of lipolysis in adipocytes, and hormonal systems. These results provide a foundation for further research into the specific functions of candidate genes encoding alternatively spliced transcripts that are differentially expressed in animals differing for REA and IF content.

## Methods

### Animals and phenotypes

The intact Nelore bulls used in this study (N = 80) were from the Capivara Farm, which participates in the Nelore Qualitas Breeding Program. The animals from a single contemporary group (that remained together from birth until slaughter) were reared on *Brachiaria* sp. and *Panicum* sp. forages, and had free access to mineral salt and were finished in a feedlot for approximately 90 days. The diet was based on whole-plant silage and mix of sorghum grain, soybean meal or sunflower seeds were used as concentrate, with a concentrate/roughage ratio from 50/50 to 70/30. The animals were slaughtered at an average age of 24 months in a commercial slaughterhouse, in accordance with Brazilian Federal Inspection Service procedures.

Samples from the *longissimus thoracis* muscle were collected, between the 12th and 13th ribs of the left half of each carcass, at two time points: immediately following slaughter for the RNA-Seq analysis (details previously described by Fonseca et al.^35^) and again at 24 h post-slaughter for the evaluation of REA and determination of IF content. REA was measured by the grid method in units of centimeters squared^71^. The Bligh and Dyer^72^ method was used to determine IF content. From the 80 animals, groups of animals were selected for RNA-Seq analysis of ribeye muscle tissue: (1) REA, the 15 animals with the highest and 15 with the lowest REA (HREA and LREA); and (2) IF, 15 animals with the highest and 15 with the lowest IF content (HIF and LIF). A Student's t-test was applied to determine if there were statistically significant differences between the selected groups (Table 2).

**Table 2:** Descriptive statistics for ribeye muscle area and intramuscular fat content of Nelore cattle.

Phenotype	N	Mean ± standard deviation	Minimum	Maximum	p-value
HREA (cm^2^)	15	83.66 ± 2.55	81	88	0.01
LREA (cm^2^)	15	65.73 ± 2.05	61	68	
HIF^a^	15	0.101 ± 0.0095	0.094	0.126	0.05
LIF^a^	15	0.063 ± 0.0049	0.051	0.067	

*HREA* high ribeye muscle area, *LREA* low ribeye muscle area, *HIF* high intramuscular fat content, *LIF* low intramuscular fat content, *N* number of animals.

^a^The % data were transformed using the arcsine square root function.

### RNA-Seq library preparation and data processing

High-quality total RNA (approximately 500 ng) extracted from the samples was processed using a TruSeq RNA Sample Preparation Kit^®^ (Illumina, San Diego, CA) according to the manufacturer's protocol. Differently bar-coded libraries were pooled to enable multiplexed sequencing and generated, on average, approximately 25 M read-pairs per sample. RNA-Seq was performed using a HiSeq 2500 System (Illumina^®^) that generated 100 bp paired-end reads.

The quality of RNA-Seq reads (quality scores, GC content, N content, length distributions, duplication, overrepresented sequences and K-mer content) was checked using FastQC (v.0.11.4) software^73^. Trimmed reads data were obtained using Trimmomatic (v.0.36)^74^, with following parameters: PE ILLUMINACLIP: TruSeq3-PE.fa:2:40:15 LEADING:20 TRAILING:20 CROP:100 SLIDINGWINDOW:4:20 AVGQUAL:20 MINLEN:50. All downstream analyses were performed on the trimmed data with high quality reads. HISAT2 (v.2.0.5)^75^ was used to align the paired-end trimmed data to the bovine reference genome (UMD3.1.1 *Bos taurus*) and chrY (*Btau* 4.6.1), both deposited in National Center for Biotechnology Information (NCBI) (https://www.ncbi.nlm.nih.gov/). The parameters used were: − p 12, − dta, − known-splicesite-infile, − x, − 1, − 2, − S. The annotation was merged both, UMC3.1.1 and chrY *(Btau* 4.6.1). Table 1 shows the descriptive statistics for the mean alignment rates for the samples by trait (REA and IF), (for detail, see “Supplementary figures”). Qualimap (v.2.2.1)^15^ was used to estimates metrics and bias, including reads genomic origin, junction analysis, transcript coverage and 5′–3′ bias computation.

### Identification of differentially expressed alternatively spliced transcripts (DAS)

JuncBASE (v.0.9) software (Junction-Based Analysis of Splicing Events)^3^ was used to identify and classify exon-centric alternative splicing events (cassette exons, alternative 5′ splice site, alternative 3′ splice site, mutually exclusive exons, coordinate cassette exons, alternative first exons, alternative last exons and intron retention) based on splice junction reads predicted using the Cufflinks2 (v.2.1.1) suite of tools (Cufflinks2, and Cuffmerge2)^76^.

For each RNA-Seq sample, the spliced alignment is given as input, in BAM format. Splice junctions passing an entropy score threshold were combined with exon coordinates from transcript reference annotations and optional novel transcripts to identify alternative splicing events. The inclusion and exclusion isoforms of each alternative splicing event are quantified using the RNA-Seq read alignments and Percentage Spliced Index (PSI) values are calculated. Differential splicing analysis is performed from isoform abundances or PSI values. For more details, the following eight steps were used^3^.

*Step 1* Building annotation database: the annotation database was used to identify all exons in the transcriptome and it was derived from a GTF file. We created two different databases. The first, was GTF file that combined Cufflinks2 and Cuffmerge2 identified transcripts with those that are annotated in a reference set, and it was used to identify all internal exons (not the first or last exon in a transcript). The second database was derived from just a reference annotation. This second database was used to define alternative first and last exons.

*Step 2* Determination of junction entropy cutoff: for all subset of samples, was built an entropy plot of annotated and novel junctions. Cutoff of 1.1 was used to reduce the number of potential false positive novel junctions.

*Step 3* BAM files processing: first, the junctions were filtered (based on entropy score obtained in step 2). From this step, for each sample, it was obtained a BED file which contained all junctions and a TXT file, which contained all the non-junction reads from the BAM file.

*Step 4* Identification of all junctions: this step created one BED file that combined junctions from all samples.

*Step 5* Building of exon–intron junction count files: this step created a counts file of the number of reads aligning to every exon–intron junction.

*Step 6* Building of pseudo/“all junction” sample: The pseudo sample was only used to define the set of all junctions and was not used for quantification. The pseudo sample was linked to the BED files created in step 4. An arbitrary sample had given in order to create other pseudo files that were not used.

*Step 7* Alternative splicing events identification and quantification of the events from each sample: this step identified, classified, and quantified alternative splicing events. The result of this step was a table with details of each alternative splicing event identified in the set of samples examined. The details of each splicing event include coordinates, and raw and length-normalized read counts to specific junctions and regions involved in the alternative splicing event. Also, we built tables of raw and length-normalized read counts of exclusion and inclusion isoforms.

*Step 8* Differential splicing analysis: this last step identified differentially spliced events between two specified groups (Highest and Lowest) of animals (REA and IF). We obtained a set of uniform transcripts for every sample in the previous steps, and then, the Percentage Spliced Index (PSI) was estimated as the ratio of the number of reads including exons to the sum of the numbers of reads including or excluding exons^3^. The PSI value was calculated and Fisher's exact test was used to test for differences in alternative splicing events between the high and low REA and IF groups (*p* < 0.05). Only alternatively spliced transcripts that were supported by at least ten reads and that had PSI differences (ΔPSI) between the respective high and low groups of greater than 5% were considered significant.

The enrichment and pathway analyses of genes set DAS transcripts associated with REA or IF content were performed using the Database for Annotation, Visualization and Integrated Discovery (DAVID v.6.8)^77^. Fisher’s exact test was used and *p values* were adjusted for multiple testing using the Benjamini–Hochberg method^78^. DAVID Pathway was used to map the gene enriched pathways using the Kyoto Encyclopedia of Genes and Genomes (KEGG) database^79^.

### Co-expression network analysis and prediction of hub genes DAS transcripts

All analyses were performed with plugins or applications from Cytoscape (v.3.7)^80^. For co-expression network analyses, the ExpressionCorrelation plugin^81^ was used to identify consensus networks of gene sets in which differentially expressed alternatively spliced transcripts were associated with REA or IF content. Similarity matrices were computed using the Pearson correlations among PSI values. A histogram tool was used for the screening criteria at node score cut-offs > 0.75 and <  − 0.75 and employed to identify statistical significance of the pairwise correlations. We established a co-expression network between the significantly co-expressed DAS genes. The cytoHubba plugin^82^ was used to explore co-expression network nodes (hub genes). The maximal clique centrality (MCC) was used for analyses, this method has a better performance on the precision of predicting essential genes from the co-expression network^78^ and to generate a subnetwork^83^. The top three hub genes were chosen to define gene co-expression subnetwork and then, it was used to analyze the functional enrichment of biological processes.

### Ethics approval

All experimental procedures were approved by Ethics Committee of the School of Agricultural and Veterinarian Sciences, São Paulo State University (UNESP), Jaboticabal, SP, Brazil (protocol number 18.340/16). The animals were provided by Qualitas Nelore breeding program company and they were slaughtered in commercial slaughterhouses. These slaughterhouses have animal welfare departments staffed by professionals trained by WAG (World Animal Protection) to ensure that the animals are killed humanely using a captive bolt pistol for stunning.

## Supplementary information

Supplementary Figures.

Supplementary Tables.

## Data Availability

The dataset utilized in this study belongs to a Qualitas Nelore breeding program company, and could be available on request. The author does not have authorization to share the data.

## References

[1] Lerch JK (2012). Isoform diversity and regulation in peripheral and central neurons revealed through RNA-Seq. PLoS ONE.

[2] Wang XG (2016). Deciphering transcriptome and complex alternative splicing transcripts in mammary gland tissues from cows naturally infected with *Staphylococcus aureus* mastitis. PLoS ONE.

[3] Brooks AN (2011). Conservation of an RNA regulatory map between drosophila and mammals. Genome Res..

[4] Guan Y, Liang G, Martin GB, Guan LL (2017). Functional changes in mRNA expression and alternative pre-mRNA splicing associated with the effects of nutrition on apoptosis and spermatogenesis in the adult testis. BMC Genomics..

[5] Nakka K, Ghigna C, Gabellini D, Dilworth FJ (2018). Diversification of the muscle proteome through alternative splicing. Skelet. Muscle..

[6] Blencowe BJ (2006). Alternative splicing: New insights from global analyses. Cell.

[7] Nilsen TW, Graveley BR (2010). Expansion of the eukaryotic proteome by alternative splicing. Nature.

[8] Sammeth M, Foissac S, Guigó R (2008). A general definition and nomenclature for alternative splicing events. Plos Comp. Biol..

[9] Potenza E (2015). Exploration of alternative splicing events in ten different grapevine cultivars. BMC Genomics..

[10] Liang G (2016). Altered microRNA expression and pre-mRNA splicing events reveal new mechanisms associated with early stage *Mycobacterium avium* subspecies paratuberculosis infection. Sci. Rep..

[11] Huang W, Khatib H (2010). Comparison of transcriptomic landscapes of bovine embryos using RNA-Seq. BMC Genomics..

[12] He H, Liu X (2013). Characterization of transcriptional complexity during longissimus muscle development in bovines using high-throughput sequencing. PLoS ONE.

[13] Scholz AM, Bünger L, Kongsro J, Baulain U, Mitchell AD (2015). Non-invasive methods for the determination of body and carcass composition in livestock: Dual-energy X-ray absorptiometry, computed tomography, magnetic resonance imaging and ultrasound: Invited review. Animal..

[14] Shingfield KJ, Bonnet M, Scollan ND (2013). Recent developments in altering the fatty acid composition of ruminant-derived foods. Animal..

[15] Konechnikov K, Conesa A, García-Alcalde F (2016). Qualimap 2: Advanced multi-sample quality control for high-throughput sequencing data. Bioinformatics.

[16] de Heredia FP, Wood IS, Trayhurn P (2010). Hypoxia stimulates lactate release and modulates monocarboxylate transporter (MCT1, MCT2, and MCT4) expression in human adipocytes. Pflugers Arch..

[17] Klein J (1990). Nomenclature for the major histocompatibility complexes of different species: A proposal. Immunogenetics.

[18] Dong J, Horvath S (2007). Understanding network concepts in modules. BMC Syst. Biol..

[19] Casci T (2006). Network fundamentals, via hub genes. Nat. Rev. Genet..

[20] Mankodi A (2003). Ribonuclear inclusions in skeletal muscle in myotonic dystrophy types 1 and 2. Ann. Neurol..

[21] Zhang L, Michal JJ (2012). Quantitative genomics of 30 complex phenotypes in Wagyu × Angus F1 progeny. Int. J. Biol. Sci..

[22] Ramos S, Khademi F, Somesh BP, Rivero F (2002). Genomic organization and expression profile of the small GTPases of the RhoBTB family in human and mouse. Gene.

[23] Attaix D (2005). The ubiquitin-proteasome system and skeletal muscle wasting. Essays Biochem..

[24] Gudjonsson T (2012). TRIP12 and UBR5 suppress spreading of chromatin ubiquitylation at damaged chromosomes. Cell.

[25] Bie AS (2016). Mutation in the HSPE1 gene encoding the mitochondrial co-chaperonin HSP10 and its potential association with a neurological and developmental disorder. Front. Mol. Biosci..

[26] Remy G (2010). Differential activation of p38MAPK isoforms by MKK6 and MKK3. Cell Signal..

[27] Bland CS (2010). Global regulation of alternative splicing during myogenic differentiation. Nucleic Acids Res..

[28] Langfelder P, Mischel PS, Horvath S (2013). When is hub gene selection better than standard meta-analysis?. PLoS ONE.

[29] Akita T (2018). De novo variants in CAMK2A and CAMK2B cause neuro developmental disorders. Ann. Clin. Trans. Neurol..

[30] Martinsen A, Dessy C, Morel N (2014). Regulation of calcium channels in smooth muscle: New insights into the role of myosin light chain kinase. Channels..

[31] Collins SP, Reoma JL, Gamm DM, Uhler MD (2000). LKB1, a novel serine/threonine protein kinase and potential tumour suppressor, is phosphorylated by cAMP-dependent protein kinase (PKA) and prenylated in vivo. Biochem. J..

[32] Salmikangas P (1999). *O. myotilin* a novel sarcomeric protein with two Ig-like domains, is encoded by a candidate gene for limb-girdle muscular dystrophy. Hum. Mol. Genet..

[33] Van der Ven PF (1999). Assignment of the human gene for endosarcomeric cytoskeletal M-protein (MYOM2) to 8p23.3. Genomics.

[34] Júnior GAF (2016). Genome scan for postmortem carcass traits in Nellore cattle. J. Anim. Sci..

[35] Fonseca LFS (2017). Differences in global gene expression in muscle tissue of Nellore cattle with divergent meat tenderness. BMC Genomics..

[36] Batra TR, Lee AJ, Gavora JS, Stear MJ (1989). CLASS I alleles of the bovine major histocompatibility system and their association with economic traits. J. Dairy Sci..

[37] Stear MJ, Pokorny TS, Muggli NE, Stone RT (1989). The relationships of birth weight, preweaning gain and postweaning gain with the bovine major histocompatibility system. J. Anim. Sci..

[38] Stupka N (2006). Activated calcineurin ameliorates contraction-induced injury to skeletal muscles of mdx dystrophic mice. J. Physiol..

[39] Fuentes JJ, Pritchard MA, Estivill X (1997). Genomic organization, alternative splicing, and expression patterns of the DSCR1 (Down syndrome candidate region 1) gene. Genomics.

[40] Sun X, Wu Y, Herculano B, Song W (2014). RCAN1 overexpression exacerbates calcium overloading-induced neuronal apoptosis. PLoS ONE.

[41] Harhay GP (2005). Characterization of 954 bovine full-CDS cDNA sequences. BMC Genomics..

[42] Hudson MB, Price SR (2013). Calcineurin: A poorly understood regulator of muscle mass. Int. J. Biochem. Cell Biol..

[43] Berdeaux R, Stewart R (2012). cAMP signaling in skeletal muscle adaptation: Hypertrophy, metabolism, and regeneration. Am. J. Physiol. Endocrinol. Metab..

[44] Chen AE, Ginty DD, Fan CM (2005). Protein kinase A signalling via CREB controls myogenesis induced by Wnt proteins. Nature.

[45] Jiang C (2010). Gene expression profiling of skeletal muscle of nursing piglets. Int. J. Biol. Sci..

[46] Quach NL, Biressi S, Reichardt LF, Keller C, Rando TA (2009). Focal adhesion kinase signaling regulates the expression of Caveolin 3 and β1 Integrin, genes essential for normal myoblast fusion. Mol. Biol. Cell..

[47] Elabd C (2014). Oxytocin is an age-specific circulating hormone that is necessary for muscle maintenance and regeneration. Nat. Comm..

[48] Holst JJ (2017). Insulin and glucagon: Partners for life. Endocrinology.

[49] Purintrapiban J, Wang MC, Forsberg NE (2003). Degradation of sarcomeric and cytoskeletal proteins in cultured skeletal muscle cells. Comp. Biochem. Physiol. B Biochem. Mol. Biol..

[50] Tidball JG, Villalta SA (2010). Regulatory interactions between muscle and the immune system during muscle regeneration. Am. J. Physiol. Regul. Integr. Comp. Physiol..

[51] Li YX (2014). Molecular cloning, sequence identification, and gene expression analysis of bovine ADCY2 gene. Mol. Biol. Rep..

[52] Veloso RC (2018). Expression of lipid metabolism and myosin heavy chain genes in pigs is affected by genotype and dietary lysine. Gen. Mol. Res..

[53] Hocquette JF (2000). Energy metabolism in skeletal muscle of meat-producing animals. INRA Prod. Anim..

[54] Sadkowski T, Jank M, Zwierzchowski L, Oprzadek J, Motyl T (2009). Transcriptomic index of skeletal muscle of beef breeds bulls. J. Physiol. Pharm..

[55] Park Y, Yoon SK, Yoon JB (2009). The HECT domain of TRIP12 ubiquitinates substrates of the ubiquitin fusion degradation pathway. J. Biol. Chem..

[56] Li QL (2010). Two novel SNPs in HSF1 gene are associated with thermal tolerance traits in Chinese Holstein cattle. DNA Cell Biol..

[57] Baena MM (2018). HSF1 and HSPA6 as functional candidate genes associated with heat tolerance in Angus cattle. R. Bras. Zootec..

[58] Deb R (2014). Effect of heat stress on the expression profile of Hsp90 among Sahiwal (*Bos indicus*) and Frieswal (*Bos indicus *× *Bos taurus*) breed of cattle: A comparative study. Gene.

[59] Ryu J (2012). Association of bovine carcass phenotypes with genes in an adaptive thermogenesis pathway. Mol. Biol. Rep..

[60] Hocquette JF (2010). Intramuscular fat content in meat-producing animals: Development, genetic and nutritional control, and identification of putative markers. Animal..

[61] Rahemi H, Nigam N, Wakeling JM (2015). The effect of intramuscular fat on skeletal muscle mechanics: Implications for the elderly and obese. J. R. Soc. Interface..

[62] Gambarota G, Janiczek RL, Mulkern RV, Newbould RD (2012). An NMR phantom mimicking intramyocellular (IMCL) and extramyocellular lipids (EMCL). Appl. Magn. Reson..

[63] Gingras AC (1999). Regulation of 4E-BP1 phosphorylation: A novel two-step mechanism. Genes Dev..

[64] Laplante M, Sabatini DM (2009). mTOR signaling at a glance. J. Cell Sci..

[65] Porstmann T (2008). SREBP activity is regulated by mTORC1 and contributes to Akt-dependent cell growth. Cell Metab..

[66] Rafalski VA, Brunet A (2011). Energy metabolism in adult neural stem cell fate. Prog. Neurobiol..

[67] Sheng X (2014). RNA-seq analysis of bovine intramuscular, subcutaneous and perirenal adipose tissues. Mol. Biol. Rep..

[68] Wei S (2015). Enhanced mitogenesis in stromal vascular cells derived from subcutaneous adipose tissue of Wagyu compared with those of Angus cattle. J. Anim. Sci..

[69] Huang W (2017). Global transcriptome analysis identifies differentially expressed genes related to lipid metabolism in Wagyu and Holstein cattle. Sci. Rep..

[70] Cesar AS (2015). Putative regulatory factors associated with intramuscular fat content. PLoS ONE.

[71] United States Department of Agriculture-USDA (1997). Official United States Standards for Grades of Carcass Beef.

[72] Bligh EG, Dyer WJ (1959). A rapid method of total lipid extraction and purification. Can. J. Biochem. Physiol..

[73] Andrews, S. FastQC: A quality control tool for high throughput sequence data [Online]. http://www.bioinformatics.babraham.ac.uk/projects/fastqc/ (2010).

[74] Bolger AM, Lohse M, Usadel B (2014). Trimmomatic: A flexible trimmer for illumina sequence data. Bioinformatics.

[75] Kim D, Langmead B, Salzberg SL (2015). HISAT: A fast spliced aligner with low memory requirements. Nat. Meth..

[76] Trapnell C (2012). Differential gene and transcript expression analysis of RNA-Seq experiments with TopHat and Cufflinks. Nat. Protoc..

[77] da Huang W, Sherman BT, Lempicki RA (2009). Systematic and integrative analysis of large gene lists using DAVID bioinformatics resources. Nat. Protoc..

[78] Benjamini Y, Hocheberg Y (1995). Controlling the false discovery rate: A practical and powerful approach to multiple testing. J. Roy. Stat. Soc..

[79] Kanehisa M, Goto S (2000). KEGG: Kyoto encyclopedia of genes and genomes. Nucleic Acids Res..

[80] Shannon P (2003). Cytoscape: A software environment for integrated models of biomolecular interaction networks. Genome Res..

[81] Hui, S. *et al.* Expression Correlation [Online]. https://apps.cytoscape.org/apps/expressioncorrelation (2015).

[82] Chin CH (2014). CytoHubba: Identifying hub objects and sub-networks from complex interactome. BMC Syst. Biol..

[83] Stevens A, De Leonibus C, Hanson D, Dowsey AW, Whatmore A, Meyer S (2013). Network analysis: A new approach to study endocrine disorders. J. Mol. Endocrinol..

