# Supplementary material for: Spliced genes in muscle from Nelore Cattle and their association with carcass and meat quality
Source: Sci Rep. 2020 Sep 7;10:14701. doi: 10.1038/s41598-020-71783-4 (PMC7477197; doi:10.1038/s41598-020-71783-4)
Supplement: Supplementary file 1 — Supplementary Figures. [file 41598_2020_71783_MOESM1_ESM.pdf]

Supplementary Figures – S1, S2, S3, S4 and S5

Spliced genes in muscle from Nelore Cattle and their association with carcass and meat quality

Danielly B. S. Silva, Larissa F. S. Fonseca, Daniel G. Pinheiro, Ana F. B. Magalhães, Maria M. M. Muniz, Jesus A. Ferro, Fernando Baldi, Luis A. L. Chardulo, Robert D. Schnabel, Jeremy F. Taylor, Lucia G. Albuquerque.

Quality control report from Qualimap (v.2.2.1)[15]

Quality control was performed with RNA-seq data (BAM file) from muscle tissue of Nelore Cattle. Basic statistics of the alignment (number of reads, coverage, GC-content, etc.) and bias estimations (reads genomic origin and junction analysis) summarized this file. For more details, all metrics for each file BAM (individually) is available for download in ZENODO (https://zenodo.org/)

DOI: 10.5281/zenodo.3939144

Link: https://zenodo.org/record/3939144#.Xwy72yhKjIU

S1. Multi-sample BAM QC analysis generated by Qualimap (v.2.2.1) for ribeye area group.

Highest ribeye area (HREA)				Lowest ribeye area (LREA)			
**Sequence ID**	**Raw reads pairs (Mb)**	**Trimmed reads pairs (Mb)**	**Overall alignment rate (%)**	**Sequence ID**	**Raw reads pairs (Mb)**	**Trimmed reads pairs (Mb)**	**Overall alignment rate (%)**
N5001	17,525,193	14,121,641	96.56	N5005	37,389,485	34,511,728	96.53
N5034	27,719,519	25,817,447	96.23	N5010	12,069,555	9,706,541	96.18
N5079	12,604,475	10,209,149	96.53	N5003	37,716,840	30,583,246	96.40
N5006	32,645,772	30,068,141	96.22	N5046	36,961,023	34,576,999	96.13
N5078	35,983,786	33,405,266	96.95	N5050	28,723,235	26,921,059	96.23
N5108	26,222,229	21,813,841	96.10	N5060	15,096,673	12,227,626	96.72
N5069	13,897,839	11,316,252	96.63	N5089	16,105,988	13,342,281	96.74
N5109	31,387,621	26,298,812	96.31	N5090	14,024,452	11,582,059	96.48
N5076	12,301,661	9,933,368	96.63	N5020	28,723,067	26,468,090	96.37
N5070	11,097,629	9,005,236	96.63	N5038	13,133,847	10,580,378	96.45
N5064	37,606,988	34,953,931	96.57	N5096	49,565,198	45,685,957	97.04
N5036	13,898,951	11,215,972	96.34	N5106	30,560,144	25,173,073	96.43
N5056	15,074,201	12,984,323	96.44	N5002	51,165,141	41,041,717	96.68
N5047	32,897,105	30,731,330	94.46	N5009	14,024,835	11,2961,32	97.12
N5039	33,068,852	30,751,981	96.29	N5049	34,107,958	27,737,678	96.82
**Mean**	23,595,455	20,841,779	96.33	**Mean**	27,957,829	24,095,638	96.55

S1.1. Descriptive statistics for alignment parameters for ribeye area group

Number of samples	30
Number of groups	2
Total number of mapped reads	1,381,962,641
Mean samples coverage	63.85
Mean samples GC-content	53.13
Mean samples mapping quality	58.7
Mean samples insert size	182.2

S1.2. The summary table contains comparison of selected critical alignment metrics for all samples selected for ribeye area trait. The metrics include mean and standard deviation of coverage, mean GC content, mean insert size and mean mapping quality.

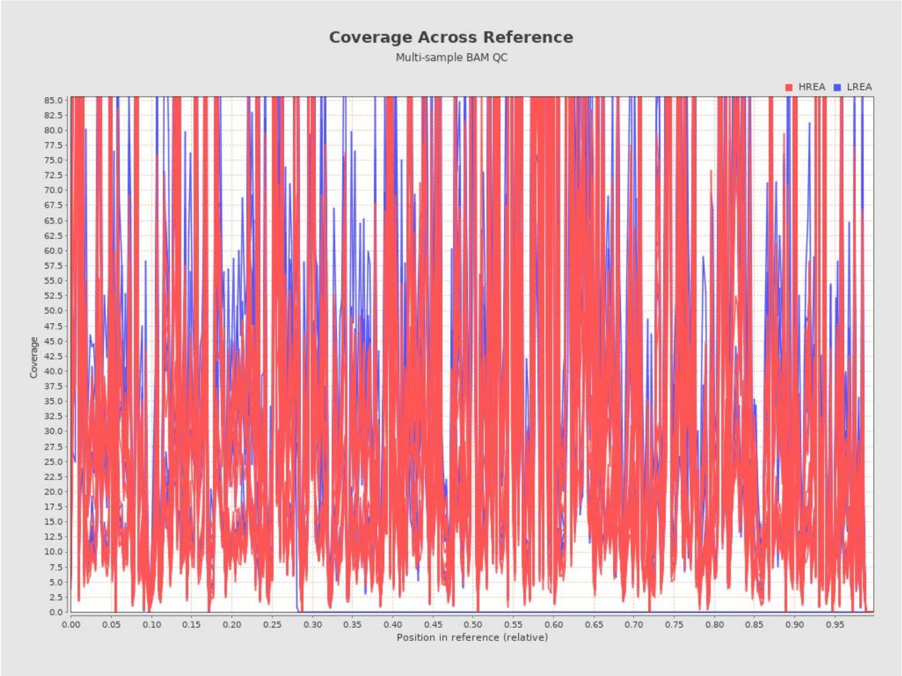

S1.3. Coverage distribution (red and blue lines) and coverage deviation across the reference sequence.

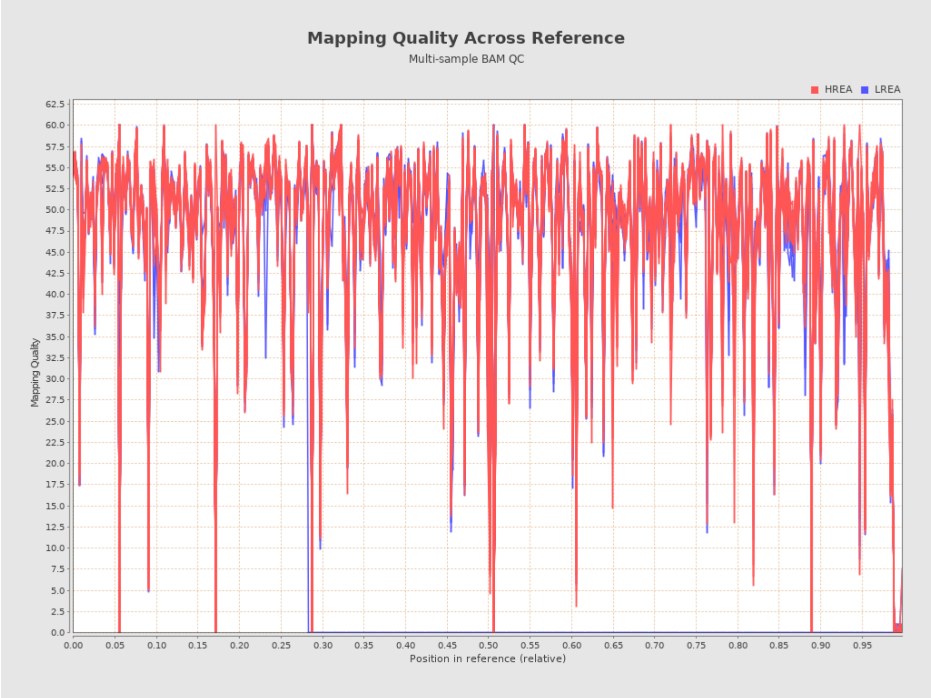

S1.4. Mapping quality distribution across the reference. To construct the plot, mean mapping quality is computed for each window.

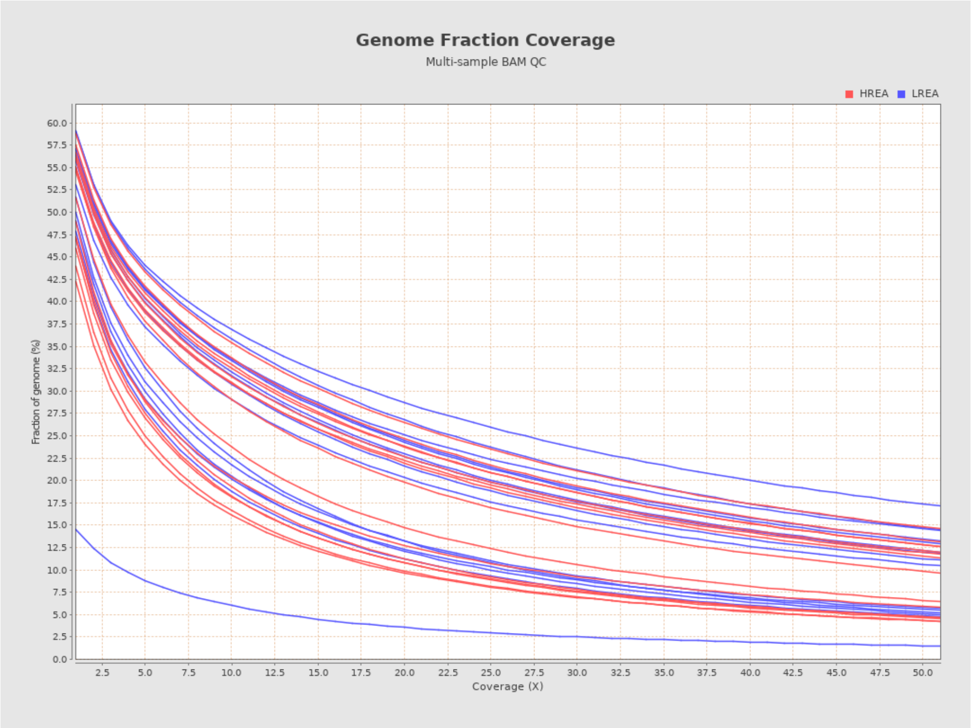

S5. Sample coverage rate in ratio to the reference genome

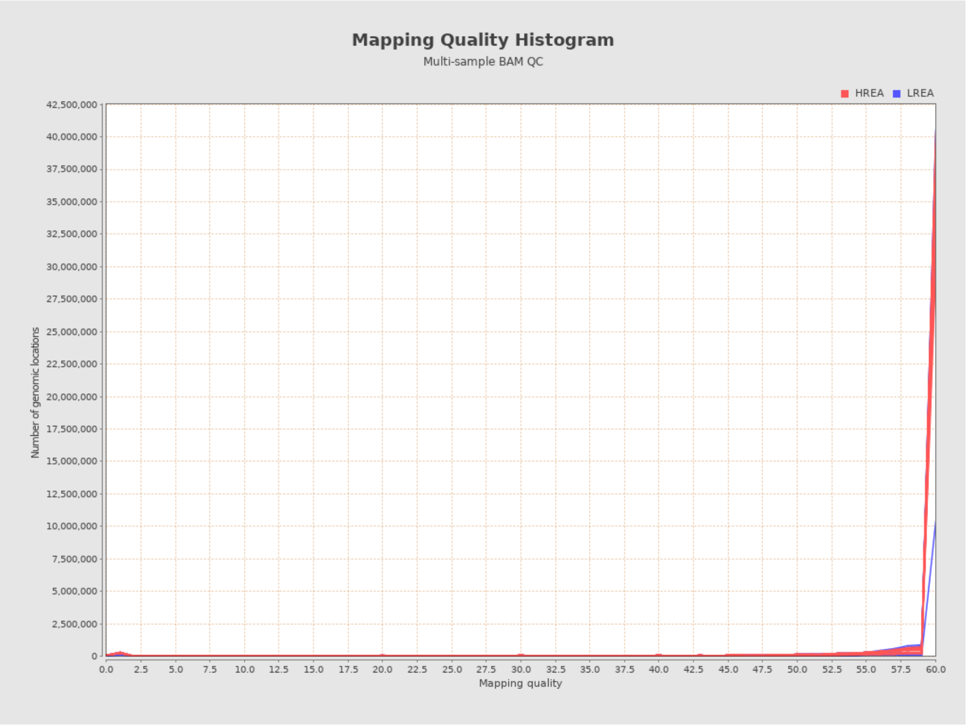

S1.6. Histogram of the number of genomic locations having a given mapping quality.

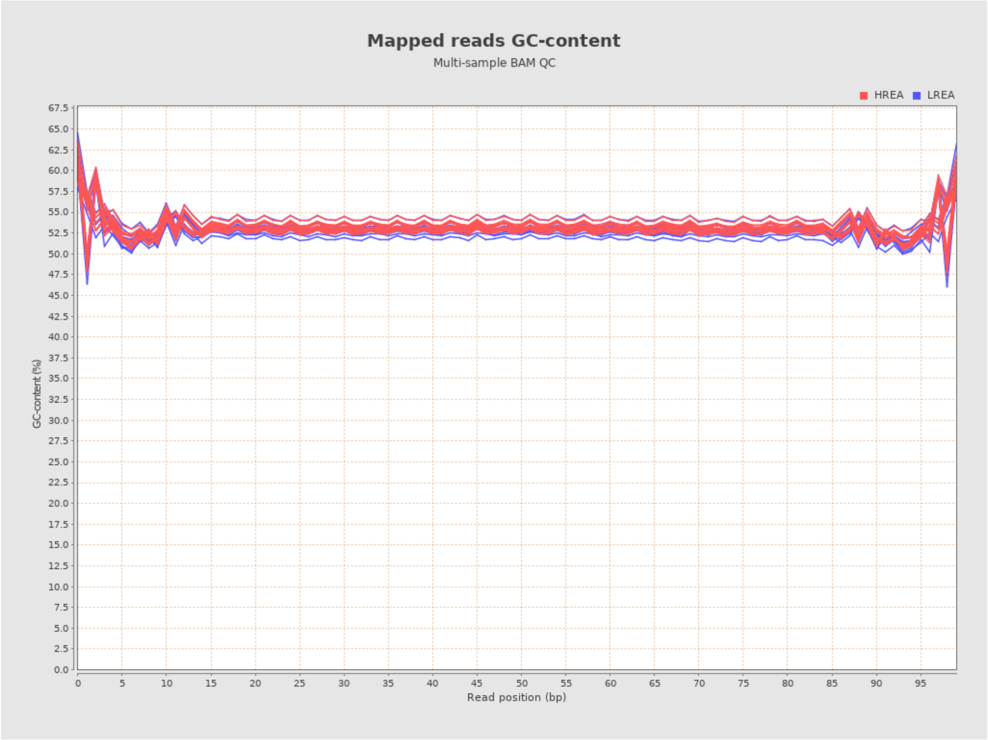

S1.7. GC content across reference together with its average value (red and blue dotted line).

S2. Multi-sample BAM QC analysis generated by Qualimap (v.2.2.1) for intramuscular fat content group.

Highest intramuscular fat content (HIF)				Lowest intramuscular fat content (LIF)			
**Sequence ID**	**Raw reads pairs (Mb)**	**Trimmed reads pairs (Mb)**	**Overall alignment rate (%)**	**Sequence ID**	**Raw reads pairs (Mb)**	**Trimmed reads pairs (Mb)**	**Overall alignment rate (%)**
N5050	28,723,235	26,921059	96.23	N5054	32,171,041	30,151,500	96.7
N5014	33,597,350	30,905727	96.62	N5010	12,069,555	9,706,541	96.18
N5006	32,645,772	30,068141	96.22	N5018	30,857,496	28,477,140	96.34
N5026	35,748,151	32,838508	96.57	N5122	14,954,299	12,425,519	96.62
N5036	13,898,951	11,215972	96.34	N5059	24,889,177	20,258,917	96.58
N5003	37,716,840	30,583246	96.4	N5094	10,183,749	8,341,290	96.38
N5103	33,083,107	30,367038	96.73	N5107	15,361,700	12,673,331	96.14
N5105	11,862,753	9,841036	96.31	N5019	31,126,455	28,771,526	96.39
N5034	27,719,519	25,817447	96.23	N5055	14,738,117	11,991,827	96.6
N5004	14,386,753	25,817447	96.23	N5088	27,567,691	22,970,733	96.55
N5115	14,790,642	11,559729	96.57	N5076	12,301,661	9,933,368	96.63
N5061	42,738,914	34,531411	96.81	N5125	13,674,494	11,290,360	95.9
N5002	51,165,141	41,041717	96.68	N5084	26,558,216	21,997,335	96.52
N5109	31,387,621	26,298812	96.31	N5038	13,133,847	10,580,378	96.45
N5029	29,626,011	27,625207	96.56	N5082	28,665,948	23,466,657	96.49
**Mean**	29,272,717	26,362166	96.454	**Mean**	20,550,230	17,535,761	96,431

S2.1. Descriptive statistics for alignment parameters for intramuscular fat content group.

Number of samples	30
Number of groups	2
Total number of mapped reads	1,405,739,187
Mean samples coverage	65.42
Mean samples GC-content	53.15
Mean samples mapping quality	58.7
Mean samples insert size	182.47

S2.2. The summary table contains comparison of selected critical alignment metrics for all samples selected for intramuscular fat content. The metrics include mean and standard deviation of coverage, mean GC content, mean insert size and mean mapping quality.

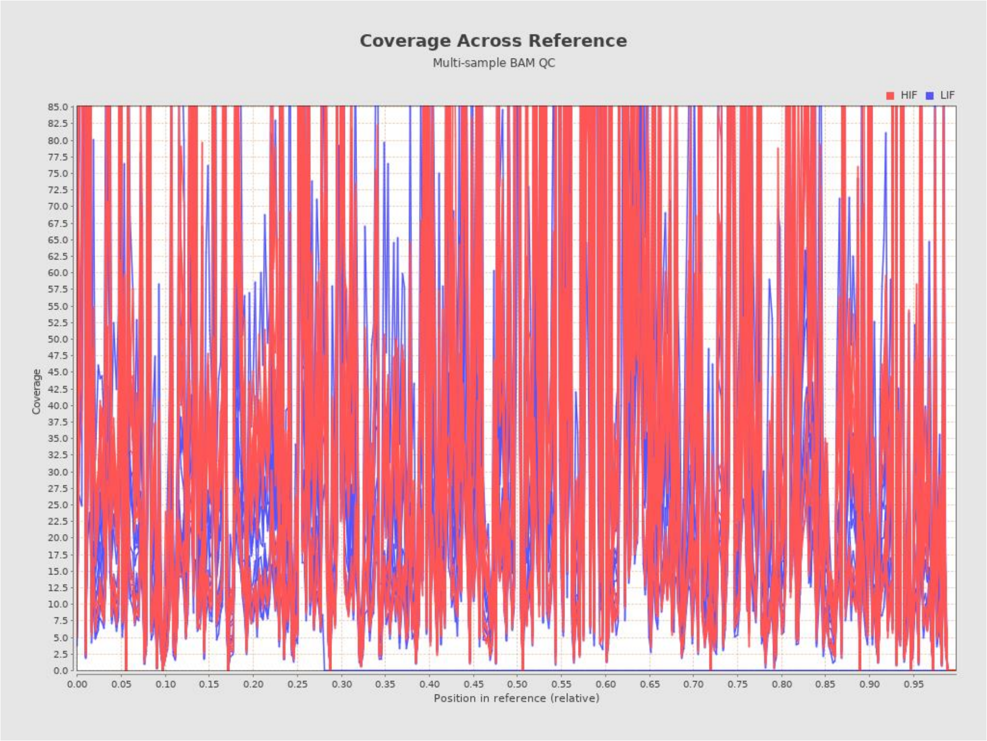

S2.3. Coverage distribution (red and blue lines) and coverage deviation across the reference sequence.

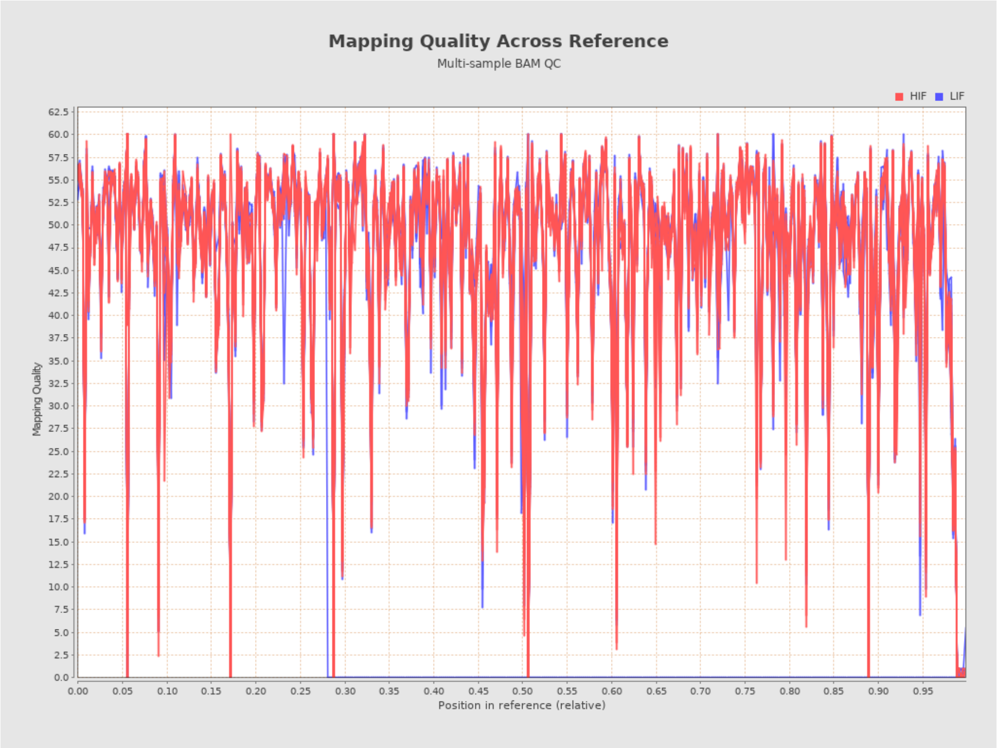

S2.4. Mapping quality distribution across the reference. To construct the plot, mean mapping quality is computed for each window.

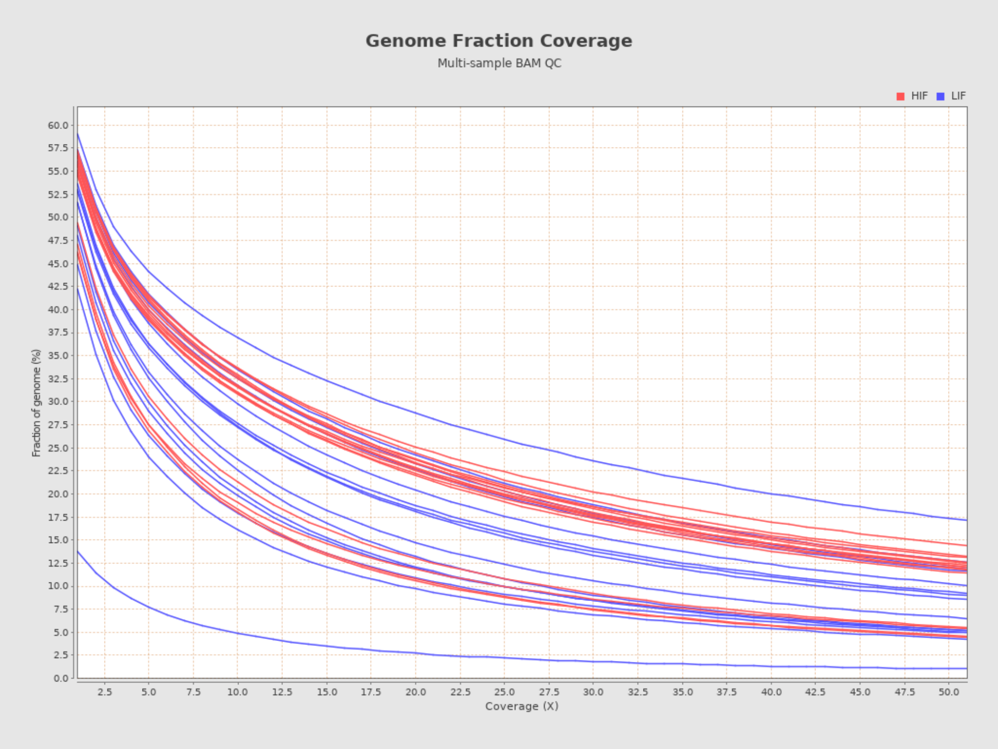

S2.5. Sample coverage rate in ratio to the reference genome

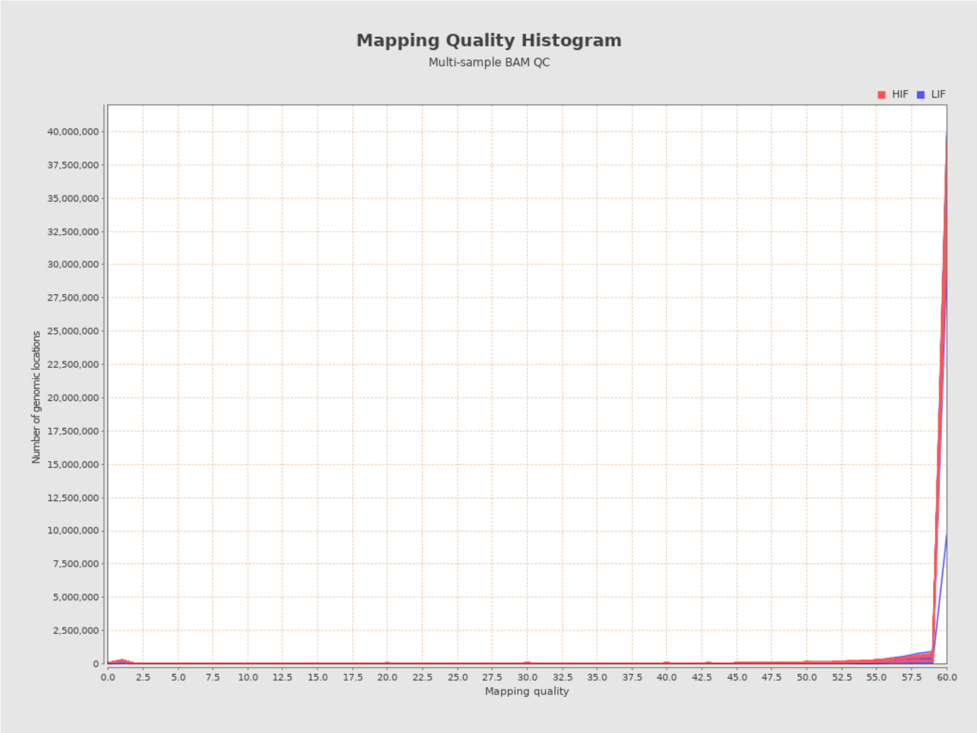

S2.6. Histogram of the number of genomic locations having a given mapping quality.

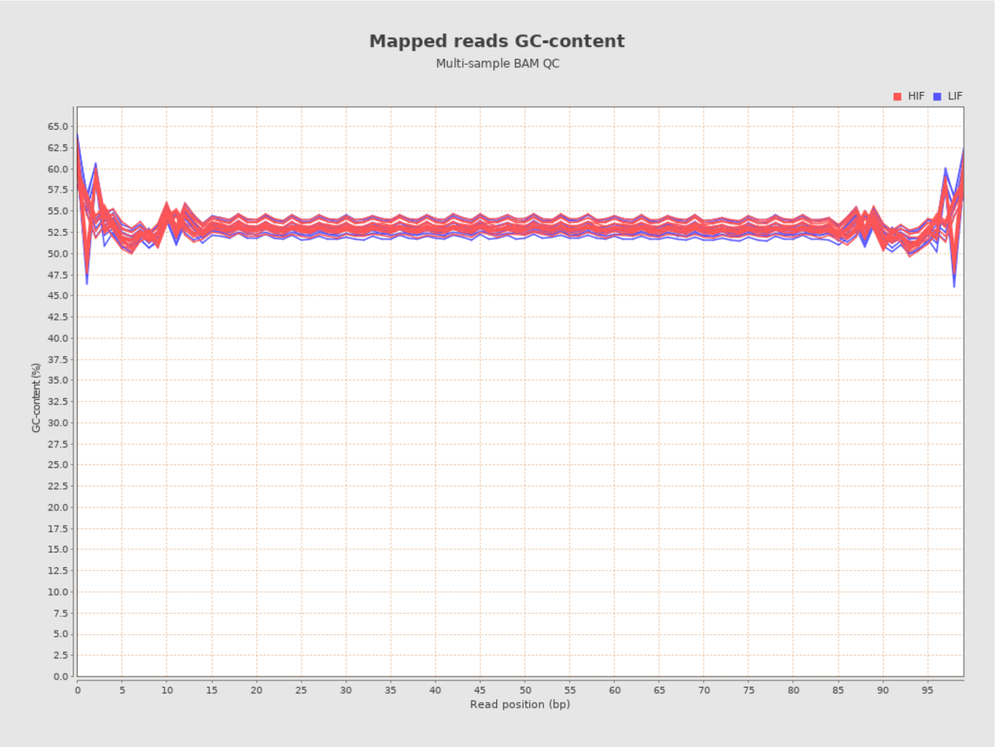

S2.7. GC content across reference together with its average value (red and blue dotted line).

S3. RNA Seq QC report summarized for ribeye area group. (Qualimap v.2.2.1)

Animal group	Sequence ID	Reads genomic origin			Reads at junctions
		Exonic	Intronic	Intergenic	Novel, known and partly known (Total)
Highest ribeye area (HREA)	N5001	16,601,728	6,261,931	3,746,355	10,208,002
	N5034	33,349,422	8,631,770	6,247,080	18,989,905
	N5079	12,123,120	4,422,599	2,437,403	7,880,438
	N5006	38,644,396	11,446,285	5,891,082	23,606,121
	N5078	41,781,550	11,421,418	9,619,191	25,242,580
	N5108	24,874,456	9,577,959	5,754,252	15,956,332
	N5069	13,223,252	5,029,364	3,084,740	8,349,804
	N5109	32,126,836	11,655,352	5,412,851	20,709,171
	N5076	12,002,474	4,252,811	2,387,644	8,025,452
	N5070	11,332,011	3,665,127	1,925,672	7,408,086
	N5064	42,864,568	12,325,307	8,939,650	25,424,953
	N5036	12,236,788	4,613,955	3,165,861	7,646,878
	N5056	15,997,961	5,700,240	2,445,162	10,687,078
	N5047	10,687,078	11,110,885	8,805,158	22,551,359
	N5039	39,582,975	11,561,301	6,315,024	23,831,655
Lowest ribeye area (LREA	N5005	42,721,005	12,200,592	9,872,883	25,537,998
	N5010	4,245,319	4,245,319	2,693,233	6,319,964
	N5003	36,699,872	13,351,837	7,025,599	23,237,805
	N5046	42,117,990	12,490,933	9,441,142	26,408,737
	N5050	33,397,302	9,100,649	7,803,269	19,764,799
	N5060	14,636,631	5,140,198	3,200,703	9,461,383
	N5089	15,629,214	5,947,683	3,613,400	9,753,557
	N5090	13,775,119	5,125,842	2,781,558	8,815,055
	N5020	33,424,198	9,922,701	6,193,708	20,226,301
	N5038	12,787,447	4,743,430	2,367,649	7,686,846
	N5096	56,867,889	16,214,582	12,689,675	33,259,503
	N5106	29,886,537	10,721,917	6,463,692	18,691,608
	N5002	48,994,826	18,169,779	9,515,245	33,584,412
	N5009	12,454,832	4,296,194	4,676,062	7,919,340
	N5049	32,206,136	11,348,029	8,670,467	20,384,321
Mean		27,393,077	9,061,391	5,404,556	16,918,981

S3.1. Descriptive statistics for genomic origin and junction read for ribeye area group

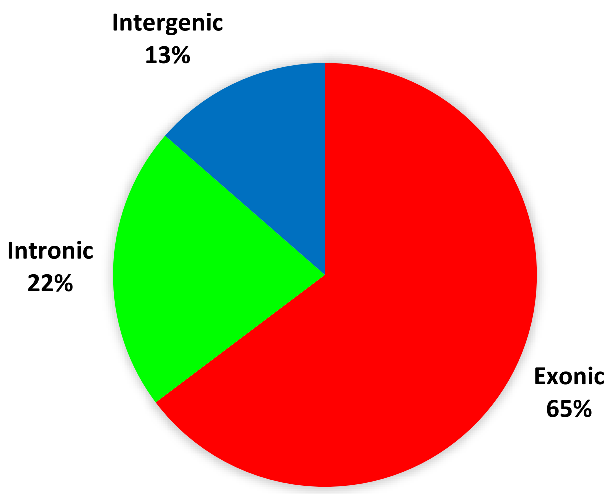

S3.2. Reads Genomic Origin for ribeye area group. Pie chart showing how many of read alignments fall into exonic, intronic and intergenic regions[15].

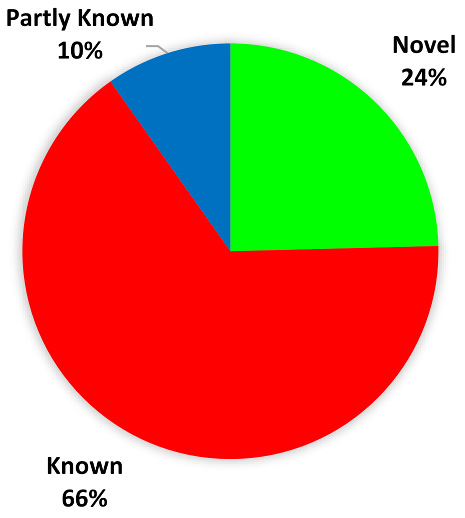

S3.3. Junction analysis for ribeye area group. This pie chart shows analysis of junction positions in spliced alignments. Known category represents percentage of alignments where both junction sides are known. Partly known represents alignments where only one junction side is known. All other alignments with junctions are marked as Novel[15].

S4. RNA Seq QC report summarized for intramuscular fat content group. (Qualimap v.2.2.1)

Animal group	Sequence ID	Reads genomic origin			Reads at junctions
		Exonic	Intronic	Intergenic	Novel, known and partly known (Total)
Highest intramuscular fat content (HIF)	N5050	33,397,302	9,100,649	7,803,269	19,764,799
	N5014	39,775,612	11,042,525	6,877,439	23,052,513
	N5006	38,644,396	11,446,285	5,891,082	23,606,121
	N5026	42,204,386	11,441,483	7,409,920	25,152,011
	N5036	12,236,788	4,613,955	3,165,861	7,646,878
	N5003	36,699,872	13,351,837	7,025,599	23,237,805
	N5103	36,160,006	10,242,444	9,156,993	21,704,032
	N5105	12,026,841	4,181,563	2,227,204	7,455,193
	N5034	33,349,422	8,631,770	6,247,080	18,989,905
	N5004	13,677,752	5,018,375	2,792,858	8,558,787
	N5115	15,456,145	5,168,263	2,501,427	10,264,529
	N5061	42,324,639	15,004,677	7,511,466	28,294,293
	N5002	48,994,826	18,169,779	9,515,245	33,584,412
	N5109	32,126,836	11,655,352	5,412,851	20,709,171
	N5029	34,206,363	9,542,990	7,858,086	20,330,764
Lowest intramuscular fat content (LIF)	N5054	37,665,204	10,687,943	8,097,577	22,891,111
	N5010	4,245,319	4,245,319	2,693,233	6,319,964
	N5018	34,925,622	10,465,584	7,575,385	21,656,093
	N5122	14,486,214	5,309,675	3,512,410	9,528,277
	N5059	23,801,241	8,866,473	5,218,160	15,571,283
	N5094	10,366,411	3,607,775	1,666,042	6,624,418
	N5107	15,225,887	5,575,384	2,743,508	9,884,630
	N5019	35,163,631	10,154,993	8,272,508	21,471,585
	N5055	14,755,859	5,426,059	2,300,104	9,888,189
	N5088	27,464,550	10,386,345	5,020,184	18,078,991
	N5076	12,002,474	4,252,811	2,387,644	8,025,452
	N5125	13,184,266	4,262,119	3,457,265	7,702,993
	N5084	26,735,363	9,892,697	4,556,927	17,875,399
	N5038	12,787,447	4,743,430	2,367,649	7,686,846
	N5082	26,934,918	10,109,576	6,404,317	17,299,509
**Mean**		23,913,085	8,542,471	5,283,959	16,428,532

S4.1. Descriptive statistics for genomic origin and junction read for intramuscular fat content group.

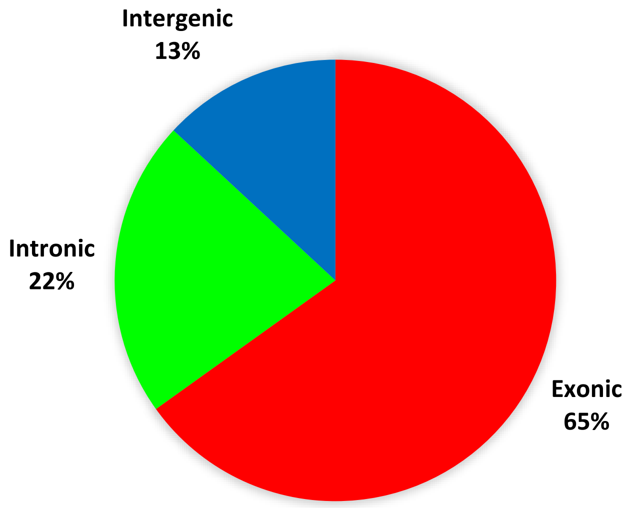

S4.2. Reads Genomic Origin for intramuscular fat content group. Pie chart showing how many of read alignments fall into exonic, intronic and intergenic regions[15].

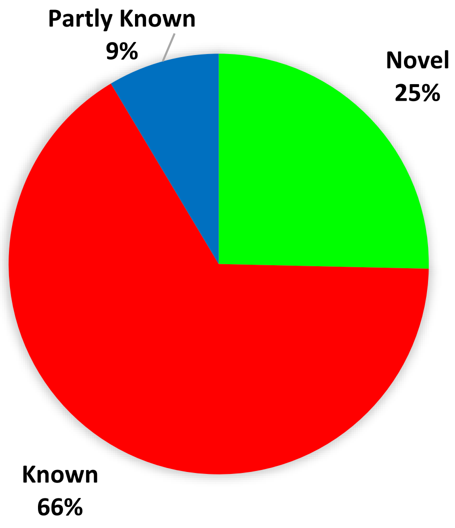

S4.3. Junction analysis for intramuscular fat content group. This pie chart shows analysis of junction positions in spliced alignments. Known category represents percentage of alignments where both junction sides are known. Partly known represents alignments where only one junction side is known. All other alignments with junctions are marked as Novel[15].

S5. RNA Seq QC report for N5050 sample. (Qualimap v.2.2.1). The comprehensive QC report for an individual sample can be accessed by clicking the corresponding sample identifier through the link: https://zenodo.org/record/3939144#.Xwy72yhKjIU

5' bias:	0.76
3' bias:	0.44
5'-3' bias:	1.43

S5.1. Transcript coverage profile for N5050 sample. The profile provides ratios between mean coverage at the 5’ region, the 3’ region and the whole transcript. The 5’ bias is the ratio between mean coverage at the 5’ region and the whole transcript, while the 3’ bias is the ratio between mean coverage at the 3’ region and the whole transcript. 5’-3’ bias is the ratio between both biases. To compute these values for each transcript, mean coverage along with mean coverage in first 100 bp (5’ region) and last 100 bp (3’region) are calculated and collected. Afterwards, the collected values are sorted and median is selected from each array to compute the ratios[15].

Reads at junctions:	19,764,799
ACCT	5.02%
AGGT	4.34%
ATCT	4.23%
GCCT	3.63%
TCCT	3.1%
AGGC	2.76%
AGCT	2.75%
AGGA	2.52%
CCCT	2.51%
CTCT	2.2%
AGAA	2.14%

S5.2. Junction analysis for N5050 sample. Total number of reads with splice junctions and 11 most frequent junction rates[15].

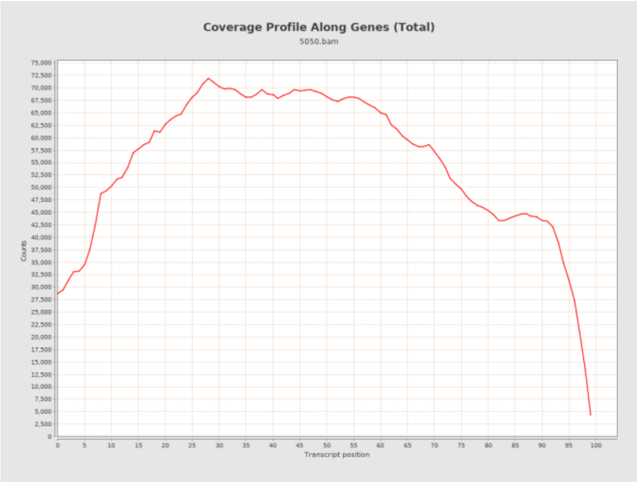

S5.3. Coverage profile along genes for N5050 sample. The plot shows mean coverage profile of the transcripts. All transcripts with non-zero coverage are used to calculate this plot[15].

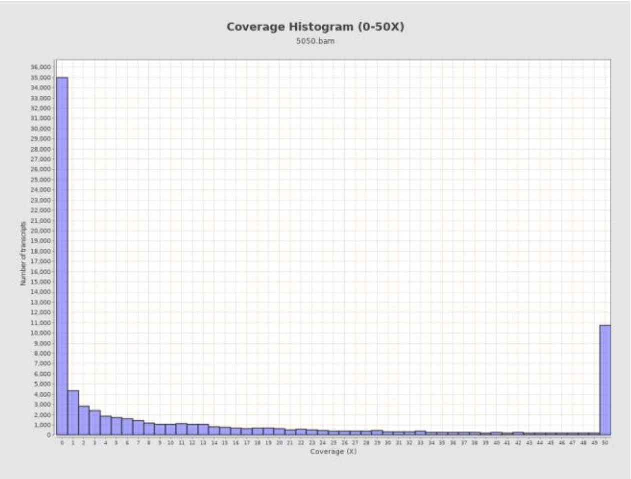

S5.4. Coverage Histogram (0-50x) for N5050 sample. Coverage of transcripts from 0 to 50X. If certain genes have higher coverage level they are added to the last column (50X) [15].
