# Supplementary material for: Spliced genes in muscle from Nelore Cattle and their association with carcass and meat quality
Source: Sci Rep. 2020 Sep 7;10:14701. doi: 10.1038/s41598-020-71783-4 (PMC7477197; doi:10.1038/s41598-020-71783-4)
Supplement: Supplementary file 2 — Supplementary Tables. [file 41598_2020_71783_MOESM2_ESM.pdf]

Supplementary Tables – S1, S2, S3 and S4

Spliced genes in muscle from Nelore Cattle and their association with carcass and meat quality

Danielly B. S. Silva, Larissa F. S. Fonseca, Daniel G. Pinheiro, Ana F. B. Magalhães, Maria M. M. Muniz, Jesus A. Ferro, Fernando Baldi, Luis A. L. Chardulo, Robert D. Schnabel, Jeremy F. Taylor, Lucia G. Albuquerque.

Table S1. Differentially expressed alternatively spliced genes ($p < 0.05$, t-test) identified in the ribeye muscle of Nelore bulls with the highest and lowest ribeye muscle area animals (HREA and LREA). Green highlighted: hub genes. HREA: Highest ribeye area. LREA: Lowest ribeye area. The set of uniform transcripts for every sample and then the Percentage Spliced Index (PSI) was estimated as the ratio of the number of reads including exons to the sum of the numbers of reads including or excluding exons. The PSI value was calculated and Fisher's exact test was used to test for differences in alternative splicing events between the high and low REA (p<0.05). Only alternatively spliced transcripts that were supported by at least 10 reads and that had PSI differences (ΔPSI) between the respective high and low groups of greater than 5% were considered significant.

**Annotated Junctions (Known and Novel)**	**Transcript type**	**Gene**	**Chr**	**Strand**	**PSI HREA**	**PSI LREA**	**ΔPSI Δ (LREA-HREA)**	***p* value**
K	cassette exon	*ACAA2*	chr24	-	100	94.74	-5.26	0.014
N	alternative 3’ splice site	*ADCY2*	chr20	-	78.26	84.21	5.95	0.033
N	alternative 3’ splice site	*ADCY2*	chr20	-	17.46	15.79	-1.67	0.043
K	alternative 5’ splice site	*ADD1*	chr6	-	10	5.26	-4.74	0.048
K	intron retention	*ADD1*	chr6	-	15.36	8	-7.36	0.038
N	alternative 3’ splice site	*AKAP1*	chr19	+	90.45	93.54	3.08	0.036
N	alternative 3’ splice site	*ANAPC11*	chr19	-	3.45	2.47	-0.98	0.025
K	cassette exon	*APLP2*	chr29	+	79.37	84.62	5.25	0.022
N	alternative 3’ splice site	*ASPH*	chr14	-	79.26	70.83	-8.43	0.013

N	alternative 3’ splice site	*BOLA-DOA*	chr23	+	97.56	91.45	-6.11	0.023
N	alternative 5’ splice site	*C23H6or*	chr23	-	71.93	64.24	-7.69	0.018
N	alternative 5’ splice site	*C23H6or*	chr23	-	27.63	35.76	8.13	0.023
N	coordinate cassette exon	*C23H6or*	chr23	-	86.21	81.13	-5.08	0.041
N	cassette exon	*C3H1orf*	chr3	+	96.36	87.5	-8.86	0.007
N	cassette exon	*C3H1orf*	chr3	+	100	90.48	-9.52	0.033
N	coordinate cassette exon	*C3H1orf*	chr3	+	100	91.67	-8.33	0.010
K	cassette exon	*C8H9orf*	chr8	+	33.69	28.33	-5.36	0.026
N	cassette exon	*CAMK2B*	chr4	+	76.92	64.19	-12.73	0.005
N	alternative 3’ splice site	*CARM1*	chr7	+	91.45	100	8.55	0.007
N	alternative 3’ splice site	*CCDC82*	chr15	+	6.67	2.94	-3.73	0.029
N	cassette exon	*CD36*	chr4	-	1.06	3.03	1.97	0.032
N	alternative 5’ splice site	*CDC34*	chr7	+	14.6	10.64	-3.96	0.018
N	alternative 5’ splice site	*CDC35*	chr7	+	76.44	79.41	2.97	0.042
K	alternative 3’ splice site	*CDV3*	chr1	-	17.5	22.78	5.28	0.026
N	alternative 3’ splice site	*CMYA5*	chr10	+	95.84	98.18	2.34	0.022
K	alternative 5’ splice site	*COPS7A*	chr5	-	11.11	15.05	3.95	0.027
K	cassette exon	*CSDE1*	chr3	+	98.29	97.18	-1.11	0.030
K	alternative last exon	*CXCL12*	chr28	+	10.99	13.79	2.8	0.016
N	alternative 5’ splice site	*DBNDD2*	chr13	+	34.84	42.47	7.62	0.043
N	alternative 5’ splice site	*DBNDD2*	chr13	+	62.83	56.23	-6.59	0.044
N	alternative 3’ splice site	*DDX39A*	chr7	+	11.76	19.44	7.68	0.035
N	cassette exon	*DR1*	chr3	-	96.77	99.08	2.31	0.049
N	intron retention	*ECHDC3*	chr13	-	91.65	95.06	3.41	0.044
N	cassette exon	*EGLN1*	chr28	+	98.82	96.38	-2.44	0.002
K	alternative 3’ splice site	*EIF4G3*	chr2	+	27.03	29.41	2.38	0.010
N	alternative 5’ splice site	*EPS15*	chr3	+	61.33	51.52	-9.81	0.004
K	alternative first exon	*FAM195A*	chr25	+	97.78	100	2.22	0.010

N	cassette exon	*FAM96B*	chr18	-	77.78	81.97	4.19	0.014
N	alternative 3’ splice site	*FLNC*	chr4	+	7.02	4.18	-2.84	0.016
N	alternative 5’ splice site	*FLNC*	chr4	+	5.57	4.53	-1.04	0.031
N	alternative 5’ splice site	*FLNC*	chr4	+	93.74	94.87	1.13	0.037
K	cassette exon	*FXR1*	chr1	-	100	97.45	-2.55	0.033
K	coordinate cassette exon	*FXR1*	chr1	-	100	97.89	-2.11	0.012
N	cassette exon	*FXYD1*	chr18	+	89.8	84.08	-5.72	0.001
N	cassette exon	*FXYD1*	chr18	+	91.94	87.62	-4.32	0.002
N	alternative 5’ splice site	*GNL3L*	chrX	+	59.18	45.65	-13.53	0.031
K	alternative 5’ splice site	*HECTD1*	chr21	-	64.11	70.45	6.34	0.005
K	cassette exon	*HNRNPD*	chr6	-	3.03	1.47	-1.56	0.024
K	alternative 5’ splice site	*HNRNPH3*	chr28	+	64.29	59.68	-4.61	0.024
K	cassette exon	*HRASLS*	chr1	-	2.44	6.76	4.32	0.046
N	alternative 5’ splice site	*HSDL2*	chr8	+	4.76	1.92	-2.84	0.009
N	alternative 3’ splice site	*HSPE1*	chr2	+	1.08	2.22	1.14	0.011
N	alternative 3’ splice site	*ITGA7*	chr5	+	37.64	28.33	-9.31	0.023
N	alternative 5’ splice site	*ITGA8*	chr5	+	38.42	30	-8.42	0.021
N	cassette exon	*KIAA0232*	chr6	+	22.07	30.7	8.63	0.035
N	alternative 5’ splice site	*KIF5B*	chr13	+	83.02	87.16	4.14	0.027
K	alternative 3’ splice site	*LIMCH1*	chr6	+	14.67	11.76	-2.91	0.031
N	alternative 3’ splice site	*LOC1008*	chr18	+	48.5	52.32	3.82	0.049
N	alternative 5’ splice site	*LOC1019*	chr4	+	0.35	2.4	2.05	0.044
N	alternative 5’ splice site	*LOC1049*	chr14	+	79.31	82.61	3.3	0.049
N	alternative 5’ splice site	*LOC104970651*	chr21	+	100	95.12	-4.88	0.025
K	alternative last exon	*LOC104972733*	chr6	+	1.61	0.7	-0.91	0.049
N	alternative 5’ splice site	*LOC7865*	chr7	-	42.11	36.21	-5.9	0.020
K	alternative first exon	*LRRC2*	chr22	+	0.75	2.67	1.92	0.047
K	alternative last exon	*LRRFIP1*	chr3	+	8.33	10.98	2.65	0.042

K	cassette exon	*LRRFIP1*	chr3	+	93.62	89.92	-3.7	0.013
K	coordinate cassette exon	*LRRFIP1*	chr3	+	64.91	81.82	16.91	0.023
N	alternative 3’ splice site	*LRRFIP2*	chr22	+	95.19	100	4.81	0.034
K	cassette exon	*LRRFIP2*	chr22	+	81.98	100	18.02	0.018
N	cassette exon	*LRRFIP2*	chr22	+	92.86	100	7.14	0.043
N	cassette exon	*MFF*	chr2	+	84.06	79.13	-4.93	0.019
N	alternative 5’ splice site	*MGLL*	chr22	+	23.2	21.1	-1.1	0.017
N	alternative 5’ splice site	*MGLL*	chr22	+	45.2	50.5	5.3	0.025
N	alternative 3’ splice site	*MGST2*	chr17	-	4.05	6.25	2.2	0.050
N	cassette exon	*MIF4GD*	chr19	+	73.53	80	6.47	0.032
N	cassette exon	*MIF4GD*	chr19	+	79.7	69.7	-10	0.038
N	cassette exon	*MLIP*	chr23	-	75	70.75	-4.25	0.021
N	cassette exon	*MLIP*	chr23	-	75	70.55	-4.45	0.024
N	coordinate cassette exon	*MLIP*	chr23	-	85.96	80.39	-5.57	0.029
N	alternative 5’ splice site	*MPRIP*	chr19	-	11.63	10.57	-1.06	0.016
K	alternative 3’ splice site	*MRPL55*	chr7	+	69.46	76.19	6.73	0.039
K	cassette exon	*MYBPC1*	chr5	+	96.34	84.55	-11.79	0.007
K	cassette exon	*MYBPC1*	chr5	+	20.88	28.65	7.77	0.015
K	cassette exon	*MYBPC1*	chr5	+	29.22	37.44	8.22	0.016
N	intron retention	*MYH1*	chr19	+	91.53	96.09	4.56	0.014
N	intron retention	*MYH1*	chr19	-	100	98	2	0.030
N	alternative 3’ splice site	*MYH1*	chr19	-	3.34	2.7	-0.64	0.009
N	alternative 3’ splice site	*MYL3*	chr22	+	96.78	100	3.22	0.046
N	alternative 5’ splice site	*MYOM2*	chr27	+	98	95.5	-2.5	0.043
N	alternative 5’ splice site	*MYOM2*	chr27	+	100	93.5	-6.5	0.048
N	cassette exon	*MYOM3*	chr2	+	100	98.91	-1.09	0.042
N	alternative 5’ splice site	*MYOT*	chr7	+	100	92	-8	0.020
N	alternative 5’ splice site	*MYOT*	chr7	+	2	8	6	0.021

N	alternative 5’ splice site	*NDRG2*	chr10	+	92.86	93.06	0.2	0.044
N	alternative 5’ splice site	*NDRG2*	chr10	+	0.96	0.38	-0.58	0.040
N	alternative 3’ splice site	*NEDD8*	chr10	+	3.17	2.17	-1	0.020
K	alternative first exon	*NTMT1*	chr11	+	3.43	5.9	-2.47	0.016
K	coordinate cassette exon	*OBSCN*	chr7	-	10.39	9.36	-1.03	0.043
N	alternative 5’ splice site	*OBSCN*	chr7	-	35.53	39.86	4.33	0.033
N	intron retention	*OBSCN*	chr7	-	100	81.82	-18.18	0.007
K	cassette exon	*OGDH*	chr4	-	23.08	29.94	6.86	0.048
N	alternative 5’ splice site	*PARK7*	chr16	-	90.32	93.24	2.92	0.010
N	alternative 5’ splice site	*PARK7*	chr16	-	8.62	6.76	-1.86	0.014
K	cassette exon	*PHLDB1*	chr15	+	6.25	12.4	6.15	0.031
N	cassette exon	*PICALM*	chr29	+	58.23	48.13	-10.1	0.048
K	alternative first exon	*PIK3R1*	chr20	-	11.11	3.98	-7.13	0.045
K	cassette exon	*PITPNA*	chr19	-	33.33	20.69	-12.64	0.034
N	alternative 3’ splice site	*PLCD4*	chr2	+	17.14	11.11	-6.03	0.007
N	alternative 3’ splice site	*PLCD4*	chr2	+	69.81	77.78	7.97	0.034
K	cassette exon	*POLR2E*	chr7	-	92	95.42	3.42	0.025
K	cassette exon	*PPP3CC*	chr8	+	83.97	77.78	-6.19	0.007
K	coordinate cassette exon	*PTPRM*	chr24	+	7.73	12.9	5.17	0.041
K	alternative first exon	*RCAN1*	chr1	+	87.5	96.68	9.18	0.017
N	alternative 3’ splice site	*RHOBTB1*	chr28	-	88.65	98.44	9.79	0.027
N	cassette exon	*RNF115*	chr3	+	100	95	-5	0.008
N	alternative 3’ splice site	*RNF157*	chr19	+	98.63	100	1.37	0.050
K	alternative 3’ splice site	*RNF181*	chr11	-	7.55	10.13	2.58	0.005
N	alternative 3’ splice site	*RPS4X*	chrX	+	96.81	95.26	-1.55	0.018
N	cassette exon	*RRAGD*	chr9	+	91.3	87.09	-4.21	0.020
N	cassette exon	*RSRC2*	chr17	+	6.9	1.08	-5.82	0.012
K	cassette exon	*SDHAF2*	chr29	+	3.64	1.69	-1.95	0.008

N	alternative 3’ splice site	*SLC25A2*	chr11	+	7.55	12.9	-5.35		0.046
N	alternative 3’ splice site	*SLC25A25*	chr11	+	100	94.2	-5.8		0.032
N	alternative 3’ splice site	*SLC29A1*	chr23	+	86.36	87.67	1.31		0.039
N	alternative 3’ splice site	*SLC29A2*	chr29	-	7.55	5.92	4.37		0.006
K	cassette exon	*SLMAP*	chr22	-	81.31	87.88	6.57		0.043
N	alternative 5’ splice site	*SMTNL1*	chr15	+	90	92.45	2.45		0.008
N	alternative 5’ splice site	*SNX3*	chr9	+	100	97.42	-2.58		0.015
K	alternative last exon	*SPECC1*	chr19	-	61.54	71.43	9.89		0.001
K	alternative last exon	*SPEG*	chr2	+	3.63	4.52	0.89		0.005
N	alternative 3’ splice site	*SPTBN1*	chr11	+	72.65	65.88	-6.77		0.037
N	alternative 3’ splice site	*SPTBN1*	chr11	+	18.18	24.14	5.96		0.008
N	alternative 5’ splice site	*SPTBN1*	chr11	+	30.43	40.24	9.81		0.004
N	alternative 3’ splice site	*SRCAP*	chr25	+	100	96	-4		0.043
K	cassette exon	*SRRM2*	chr25	-	84.96	89.86	4.9		0.035
K	cassette exon	*ST6GALN*	chr11	-	75	84.7	9.7		0.019
N	alternative 5’ splice site	*STAT3*	chr19	-	4.55	7.14	2.59		0.001
K	coordinate cassette exon	*STX8*	chr19	-	40	47.11	7.11		0.027
N	alternative 3’ splice site	*SVIL*	chr13	-	94.74	100	5.26		0.005
N	alternative 3’ splice site	*SVIL*	chr13	-	93.2	100	6.8		0.010
N	alternative 3’ splice site	*TACC2*	chr26	+	95.31	89.92	-5.39		0.036
N	alternative 3’ splice site	*TAX1BP*	chr4	-	2.38	4.67	2.29		0.016
N	alternative 3’ splice site	*TAX1BP*	chr4	-	97.3	94.91	-2.39		0.018
K	cassette exon	*TMED2*	chr17	-	82.61	77.42	-5.19		0.020
N	alternative 5’ splice site	*TMEM120*	chr25	-	16.03	11.24	-4.79		0.017
N	alternative 5’ splice site	*TMEM120*	chr25	-	83.71	88.24	4.53		0.034
N	alternative 5’ splice site	*TMEM126A*	chr29	-	8.54	5.26	-3.28		0.002
N	alternative 5’ splice site	*TMEM126A*	chr29	-	89.47	94.39	4.92		0.000
N	cassette exon	*TMEM14C*	chr23	-	41.69	47.66	5.97		0.007

N	cassette exon	*TMEM14C*	chr23	-	45.74	52.27	6.53	0.008
N	coordinate cassette exon	*TMEM14C*	chr23	-	43.52	50	6.48	0.005
N	alternative 5’ splice site	*UBR5*	chr14	+	50.5	62.9	12.14	0.038
K	alternative first exon	*UGP2*	chr11	+	12.66	10.64	-2.02	0.023
K	alternative first exon	*UGP2*	chr11	+	30.67	38.36	7.69	0.027
K	cassette exon	*USP28*	chr15	-	97.9	100	2.1	0.039
K	cassette exon	*USP28*	chr15	-	96.3	100	3.7	0.039
N	coordinate cassette exon	*USP34*	chr11	-	78.17	85.16	6.99	0.008
K	alternative 3’ splice site	*VCL*	chr28	+	71.43	67.43	-4	0.031
N	alternative 3’ splice site	*VLDLR*	chr8	-	100	96.2	-3.8	0.028
N	cassette exon	*WNK1*	chr5	+	42.97	35.63	-7.34	0.047
N	alternative 3’ splice site	*WNK2*	chr8	+	75	84.7	9.7	0.031
K	alternative first exon	*ZBTB7B*	chr3	-	82.84	89.66	6.82	0.019
N	alternative last exon	*ZFAND2B*	chr2	+	55	59.49	4.49	0.037
N	alternative 3’ splice site	*ZFAND5*	chr8	-	85.05	80.39	-4.66	0.019
N	alternative 3’ splice site	*ZFAND5*	chr8	-	2.22	3.66	1.44	0.041
K	cassette exon	*ZNF106*	chr10	-	8.46	6.25	-2.21	0.012

Table S2. Biological process Gene ontology (GO) terms and pathways enriched for genes with differentially expressed alternatively spliced transcripts in ribeye muscle in Nelore bulls selected to be divergent for ribeye muscle area.

**Category**	**Terms**	**Genes**	**p-*value***
Biological Process	GO:0042787~protein ubiquitination involved in ubiquitin-dependent protein catabolic process	*RNF115, UBR5, ANAPC11, RNF181, HECTD1*	0.01
	GO:0006936~muscle contraction	*MYOM2, MYBPC1, SLMAP*	0.02
	GO:0014823~response to activity	*SLC25A25, SMTNL1*	0.03
	GO:0032091~negative regulation of protein binding	*GNL3L, CARM1, PARK7*	0.03
	GO:0031647~regulation of protein stability	*USP28, GNL3L, ASPH*	0.04

	GO:2000021~regulation of ion homeostasis	*WNK1*, *WNK2*	0.04
	GO:0090073~positive regulation of protein homodimerization activity	*GNL3L*, *PARK7*	0.04
	GO:0033234~negative regulation of protein sumoylation	*GNL3L*, *PARK7*	0.04
	GO:0048745~smooth muscle tissue development	*ZFAND5*, *ITGA8*	0.05
	GO:0048268~clathrin coat assembly	*EPS15*, *PICALM*	0.05
	GO:0008283~cell proliferation	*USP28*, *PICALM*, *STAT3*, *TACC2*	0.06
	GO:2000637~positive regulation of gene silencing by miRNA	*STAT3*, *FXR1*	0.06
	GO:0000209~protein polyubiquitination	*RNF115*, *UBR5*, *RNF181*	0.08
Pathways	bta04921:Oxytocin signaling pathway	*ADCY2*, *PPP3CC*, *CAMK2B*, *RCAN1*, *PIK3R1*	0.01
	bta05414:Dilated cardiomyopathy	*YL3*, *ITGA8*, *ITGA7*	0.01
	bta04066:HIF-1 signaling pathway	*EGLN1*, *CAMK2B*, *STAT3*, *PIK3R1*	0.01
	bta04114:Oocyte meiosis	*ADCY2*, *PPP3CC*, *CAMK2B*, *ANAPC11*	0.02
	bta04510:Focal adhesion	*ITGA8*, *ITGA7*, *FLNC*, *PIK3R1*, *VCL*	0.02
	bta04120:Ubiquitin mediated proteolysis	*UBR5*, *RHOBTB1*, *ANAPC11*, *CDC34*	0.03
	bta04923:Regulation of lipolysis in adipocytes	*ADCY2*, *MGLL*, *PIK3R1*	0.03
	bta04261:Adrenergic signaling in cardiomyocytes	*ADCY2*, *MYL3*, *CAMK2B*, *PIK3R1*	0.03
	bta05166:HTLV-I infection	*ADCY2*, *BOLA-DOA*, *PPP3CC*, *ANAPC11*, *PIK3R1*	0.04
	bta05410:Hypertrophic cardiomyopathy (HCM)	*MYL3*, *ITGA8*, *ITGA7*	0.06
	bta04062:Chemokine signaling pathway	*ADCY2*, *CXCL12*, *STAT3*, *PIK3R1*	0.06
	bta04020:Calcium signaling pathway	*ADCY2*, *PLCD4*, *PPP3CC*, *CAMK2B*	0.07
	bta04512:ECM-receptor interaction	*CD36*, *ITGA8*, *ITGA7*	0.07
	bta04914:Progesterone-mediated oocyte maturation	*ADCY2*, *ANAPC11*, *PIK3R1*	0.07
	bta04024:cAMP signaling pathway	*FXYD1*, *ADCY2*, *CAMK2B*, *PIK3R1*	0.08
	bta05205:Proteoglycans in cancer	*CAMK2B*, *FLNC*, *STAT3*, *PIK3R1*	0.08
	bta04922:Glucagon signaling pathway	*ADCY2*, *PPP3CC*, *CAMK2B*	0.08
	bta04810:Regulation of actin cytoskeleton	*ITGA8*, *ITGA7*, *PIK3R1*, *VCL*	0.09
	bta04750:Inflammatory mediator regulation of TRP channels	*ADCY2*, *CAMK2B*, *PIK3R1*	0.10

Table S3. Differentially expressed alternatively spliced genes ($p < 0.05$) identified in the ribeye muscle of Nelore bulls with the highest and lowest intramuscular fat content animals (HIF and LIF). Green highlighted: hub genes. HIF: Highest intramuscular fat content. LIF: Lowest intramuscular fat content. The set of uniform transcripts for every sample and then the Percentage Spliced Index (PSI) was estimated as the ratio of the number of reads including exons to the sum of the numbers of reads including or excluding exons. The PSI value was calculated and Fisher's exact test was used to test for differences in alternative splicing events between the high and low IF ($p<0.05$). Only alternatively spliced transcripts that were supported by at least 10 reads and that had PSI differences (ΔPSI) between the respective high and low groups of greater than 5% were considered significant.

**Annotated Junctions (Known and Novel)**	**Transcript type**	**Gene**	**Chr**	**Strand**	**PSI HIF**	**PSI LIF**	**ΔPSI Δ (LIF-HIF)**	***p* value**
N	alternative 3’ splice site	*ACAT1*	chr15	+	98.4	100	1.6	0.024
K	cassette exon	*ACTR3B*	chr4	+	3.7	6.15	-2.45	0.021
N	cassette exon	*ACTR3B*	chr4	+	100	97.2	-2.8	0.025
N	alternative 3’ splice site	*ADSSL1*	chr21	+	92.56	100	7.44	0.020
N	intron retention	*AHNAK*	chr29	+	98	100	2	0.013
N	intron retention	*AHNAK*	chr29	+	95.4	100	4.6	0.031
K	cassette exon	*AKIRIN2*	chr9	+	97.83	100	2.17	0.039
N	alternative 5’ splice site	*AKTIP*	chr18	-	3.77	2,30	-1.47	0.032
N	alternative 5’ splice site	*ALPK2*	chr24	-	9.09	12.2	3.11	0.045
N	alternative 3’ splice site	*ANAPC11*	chr19	-	3.7	2.38	-1.32	0.007
K	alternative 5’ splice site	*ANK3*	chr28	-	95.65	97.8	2.15	0.026
N	cassette exon	*ANKRD10*	chr12	-	80.03	85.71	5.68	0.011
N	alternative 3’ splice site	*ANXA11*	chr28	+	7.41	5.41	-2	0.031
K	cassette exon	*APLP2*	chr29	+	65.69	53.85	-11.84	0.018
N	alternative 5’ splice site	*APOBEC2*	chr23	+	3.04	7.3	4.26	0.004
N	alternative 5’ splice site	*APOBEC2*	chr23	+	96.3	99.09	2.79	0.005
N	alternative 5’ splice site	*ARFGAP2*	chr15	-	3.39	7.2	3.81	0.012
N	alternative 3’ splice site	*ARHGEF10L*	chr2	-	100	94.8	-5.2	0.043
N	alternative 3’ splice site	*ARIH2*	chr22	-	94.67	100	5.33	0.050

K	alternative first exon	*ARMCX3*	chrX	+	15.91	11.11	-4.8	0.030
N	alternative 3’ splice site	*ARPP21*	chr22	+	9.52	4.4	-5.12	0.032
K	alternative first exon	*ART4*	chr6	+	15.59	13.96	-1.62	0.041
N	cassette exon	*ART4*	chr6	+	83.33	89.86	6.53	0.034
K	cassette exon	*ATL2*	chr11	-	98.4	100	1.6	0.019
K	alternative 3’ splice site	*ATN1*	chr5	-	13.64	15.84	2.2	0.040
N	alternative 5’ splice site	*ATP6V1C*	chr14	-	2.86	1.2	-1.66	0.025
N	cassette exon	*ATXN10*	chr5	+	96.83	100	3.17	0.017
N	alternative 3’ splice site	*AURKAIP*	chr16	+	15.65	8.12	-7.53	0.038
N	alternative first exon	*BCCIP*	chr26	+	100	98	-2	0.011
K	coordinate cassette exon	*BIN1*	chr2	+	97.92	100	2.08	0.048
K	intron retention	*BUD31*	chr25	-	5.41	2.56	-2.85	0.042
N	alternative 3’ splice site	*C10H15o*	chr10	-	8.77	3.85	-4.92	0.011
N	alternative 5’ splice site	*C10H15o*	chr10	-	15.79	10.1	-5.69	0.006
N	alternative 3’ splice site	*C13H20o*	chr13	+	25	29.29	4.29	0.006
N	alternative 3’ splice site	*C13H20o*	chr13	+	72.41	67.54	-4.87	0.050
K	cassette exon	*C28H10o*	chr28	+	22.22	13.64	-8.58	0.046
K	cassette exon	*C3H1orf*	chr3	+	94.03	90.38	-3.65	0.030
N	alternative 3’ splice site	*CCDC23*	chr3	+	96.5	100	3.5	0.039
K	alternative last exon	*CD58*	chr3	+	88.56	91.29	2.72	0.045
N	intron retention	*CKM*	chr18	-	76.78	83.87	7.09	0.020
K	cassette exon	*CLIP1*	chr17	+	96.61	100	3.39	0.009
N	cassette exon	*CMC1*	chr22	+	100	95.83	-4.17	0.043
N	cassette exon	*CMC2*	chr18	-	95.65	97.8	2.15	0.044
N	alternative 3’ splice site	*CMYA5*	chr10	+	96.15	100	3.85	0.015
K	alternative last exon	*COL6A2*	chr1	+	14.72	15.79	1.07	0.040
N	alternative 5’ splice site	*COPS5*	chr14	-	94.74	95.83	1.09	0.042
N	alternative 3’ splice site	*COX7C*	chr7	+	93.87	97.4	3.53	0.017

K	alternative first exon	*CS*	chr5	+	2.2	1.2	-1	0.007
K	alternative last exon	*CTNNB1*	chr22	+	79.41	83.33	3.92	0.031
N	cassette exon	*CTSD*	chr29	-	97.11	100	2.89	0.010
K	alternative 5’ splice site	*CTTN*	chr29	+	13.33	9.68	-3.65	0.019
N	cassette exon	*CUTC*	chr26	+	96.72	100	3.28	0.010
N	alternative 3’ splice site	*CUTC*	chr26	+	100	91	-9	0.048
N	alternative 3’ splice site	*DDX39B*	chr23	+	7.14	2	-5.14	0.004
N	alternative 3’ splice site	*DDX39B*	chr23	+	90.7	100	9.3	0.002
N	alternative 3’ splice site	*DES*	chr2	+	83.33	89.86	6.53	0.042
N	alternative 3’ splice site	*DES*	chr2	+	96	100	4	0.042
N	alternative 5’ splice site	*DLGAP4*	chr13	+	1.85	0.3	-1.55	0.005
N	alternative 3’ splice site	*DNM2*	chr7	+	11.54	6.51	-5.03	0.007
N	alternative 3’ splice site	*DNM2*	chr7	+	71.43	81.17	9.74	0.017
N	cassette exon	*DTNA*	chr24	-	15.59	13.96	-1.62	0.049
K	alternative first exon	*DTNA*	chr24	-	95.65	100	4.35	0.003
K	cassette exon	*DUSP26*	chr27	-	6.94	3.92	-3.02	0.008
K	cassette exon	*DYRK1B*	chr18	-	47.37	43.17	-4.2	0.017
N	alternative 3’ splice site	*EIF4B*	chr5	-	94.22	100	5.78	0.023
K	cassette exon	*EIF4G1*	chr1	-	88.24	85	-3.24	0.027
N	cassette exon	*FAM96B*	chr18	-	78.62	82.35	3.73	0.038
N	alternative 3’ splice site	*FBXW5*	chr11	+	2.5	0.5	-2	0.019
N	alternative 3’ splice site	*FBXW5*	chr11	+	97	100	3	0.018
N	alternative 3’ splice site	*FH*	chr16	+	100	98.2	-1.8	0.050
N	cassette exon	*FIBP*	chr29	-	100	93.9	-6.1	0.049
N	alternative 3’ splice site	*FKBP5*	chr23	-	97.92	100	2.08	0.047
N	alternative 3’ splice site	*FKBP5*	chr23	-	100	94	-6	0.047
N	cassette exon	*FKBP5*	chr23	-	100	92.7	-7.3	0.048
N	alternative 3’ splice site	*FLNC*	chr4	+	98.3	100	1.7	0.017

N	alternative 5’ splice site	*GALNT11*	chr4	+	3.58	2	-0.58	0.002
N	alternative 3’ splice site	*GBAS*	chr25	+	90.7	100	9.3	0.043
K	cassette exon	*GDE1*	chr25	-	4.17	2	-2.17	0.003
K	cassette exon	*GKAP1*	chr8	-	96.59	100	3.41	0.022
K	cassette exon	*GLRX2*	chr16	+	9.09	5.5	-3.58	0.002
N	alternative 5’ splice site	*GLRX2*	chr2	+	97.92	100	2.08	0.005
N	alternative 5’ splice site	*GLRX5*	chr21	+	100	97.83	-2.17	0.026
N	alternative 5’ splice site	*GLRX5*	chr21	+	100	94.8	-5.2	0.045
N	alternative 3’ splice site	*GNB2*	chr25	-	100	97.2	-2.8	0.045
N	alternative 3’ splice site	*HDLBP*	chr3	-	2.2	1.2	-1	0.047
N	alternative first exon	*HHATL*	chr22	-	2.41	6.2	3.79	0.005
N	alternative 5’ splice site	*HNRNPA1*	chr5	-	11.02	8.65	-2.37	0.034
K	cassette exon	*HNRNPDL*	chr6	-	95.65	98.98	3.33	0.020
N	alternative 5’ splice site	*HNRNPH1*	chr7	+	1.19	3.57	2.38	0.006
N	alternative 5’ splice site	*HNRNPH1*	chr7	+	14.29	8.11	-6.18	0.019
N	alternative 5’ splice site	*HNRNPH3*	chr28	+	3.49	2	-1.49	0.004
K	alternative first exon	*HNRNPK*	chr8	-	23.33	28.57	5.24	0.029
N	alternative 5’ splice site	*HSD17B8*	chr23	+	5.8	8.7	2.9	0.019
N	alternative 5’ splice site	*HSDL2*	chr8	+	4.55	1.89	-2.66	0.001
K	alternative 3’ splice site	*HSF1*	chr14	-	8.11	7.02	-1.09	0.023
N	alternative 5’ splice site	*HSP90AB1*	chr23	+	96.72	100	3.28	0.008
N	alternative 5’ splice site	*HSP90AB1*	chr23	+	3.28	1.02	-4.3	0.009
N	alternative 5’ splice site	*HSPE1*	chr2	+	2.2	1.7	-0.5	0.006
N	alternative 3’ splice site	*IDH3B*	chr13	+	91.03	93.1	2.07	0.021
N	alternative 3’ splice site	*IDH3B*	chr13	+	2.88	1.59	-1.29	0.015
N	cassette exon	*INPPL1*	chr15	+	94.28	100	5.72	0.002
N	alternative 5’ splice site	*ISOC2*	chr18	+	8.33	5.33	-3	0.043
N	intron retention	*JTB*	chr3	+	97.87	100	2.13	0.013

K	cassette exon	*KARS*	chr18	-	17.65	14.29	-3.36	0.016
N	alternative 5’ splice site	*KDM1A*	chr2	-	2.85	1,86	-1.84	0.030
N	cassette exon	*KIAA0368*	chr8	-	97.83	100	2.17	0.014
N	alternative 3’ splice site	*KIAA1671*	chr17	+	8.11	7.02	-1.09	0.037
N	alternative 5’ splice site	*KIAA1715*	chr2	+	23.4	30.43	7.03	0.022
K	cassette exon	*KIF1B*	chr16	-	96.48	100	3.52	0.031
K	intron retention	*KLF2*	chr7	-	5.17	2,20	-2.97	0.024
N	alternative 3’ splice site	*KLHL30*	chr3	-	1.83	5.26	3.42	0.019
N	alternative 3’ splice site	*KLHL30*	chr3	-	1.7	1.05	-0.65	0.021
N	alternative 3’ splice site	*KLHL30*	chr3	-	2.07	5.26	3.19	0.023
K	cassette exon	*LAMTOR2*	chr3	-	93.44	90	-3.44	0.013
N	alternative 3’ splice site	*LARP1*	chr7	+	100	89.47	-10.53	0.021
K	alternative first exon	*LINGO1*	chr21	-	9.43	4.82	-4.61	0.007
K	coordinate cassette exon	*LMO7*	chr12	+	6.06	1,01	-5.05	0.002
N	alternative 3’ splice site	*LMOD3*	chr22	+	2.94	1.79	-1.15	0.013
N	alternative 3’ splice site	*LMOD3*	chr22	+	97.06	98.22	1.16	0.015
N	cassette exon	*LOC1008*	chr10	+	92.11	100	7.89	0.033
N	alternative 3’ splice site	*LOC1019*	chr13	+	17.78	10	-7.78	0.009
N	alternative 3’ splice site	*LOC104975822*	chr25	+	2.67	1,66	-1.01	0.026
N	cassette exon	*LRRC2*	chr22	+	96.1	100	3.9	0.037
N	alternative 3’ splice site	*MAF1*	chr14	-	81.48	86.84	5.36	0.030
K	alternative first exon	*MAP2K6*	chr19	-	48.81	62.16	13.35	0.050
N	cassette exon	*MAZ*	chr25	-	36.08	32.26	-3.82	0.031
N	cassette exon	*MBNL1*	chr1	-	3.35	1,34	-1.01	0.014
N	alternative 3’ splice site	*MEF2C*	chr7	-	5.43	8.33	2.9	0.005
K	mutually exclusive exons	*MEF2D*	chr3	+	15.38	19.35	3.97	0.039
N	coordinate cassette exon	*MEMO1*	chr11	-	96.3	100	3.7	0.019
N	alternative first exon	*METTL21B*	chr5	-	95.8	100	4.2	0.003

N	alternative 5’ splice site	*MGAT4B*	chr7	+	1.7	1.05	-0.65	0.033
N	alternative 5’ splice site	*MID1IP1*	chrX	-	100	82	18,00	0.045
N	cassette exon	*MIF4GD*	chr19	+	80	67.71	-12.29	0.006
N	intron retention	*MIR208A*	chr10	+	79.9	100	20.1	0.036
N	alternative 5’ splice site	*MIR208B*	chr10	+	10.3	10.74	0.44	0.034
K	cassette exon	*MKNK2*	chr7	+	2.78	2.17	-0.61	0.020
N	cassette exon	*MLIP*	chr23	-	84.81	94.12	9.31	0.008
N	alternative 3’ splice site	*MPRIP*	chr19	-	0.91	2.27	1.36	0.049
N	cassette exon	*MPRIP*	chr19	-	51.65	58.14	6.49	0.018
N	alternative 5’ splice site	*MRPL33*	chr11	-	87.5	90	2.5	0.023
N	cassette exon	*MRPS18A*	chr23	-	95.69	100	4.31	0.008
K	cassette exon	*MRPS25*	chr22	+	92.31	96.08	3.77	0.016
N	alternative 5’ splice site	*MVP*	chr25	-	4	3	-1	0.048
N	alternative 3’ splice site	*MYH7B*	chr13	+	11.67	15.91	4.24	0.014
N	alternative 5’ splice site	*MYH7B*	chr13	+	23.4	30.43	7.03	0.006
N	alternative 3’ splice site	*MYH7B*	chr13	+	80	72.73	-7.27	0.023
N	alternative 3’ splice site	*MYLK2*	chr3	-	10.45	7.14	-3.31	0.028
N	alternative 3’ splice site	*MYLK2*	chr3	-	3.85	1.69	-2.16	0.032
N	alternative 3’ splice site	*MYLK2*	chr3	-	13.55	8	-5.55	0.038
N	intron retention	*MYO18A*	chr19	-	12.63	6.67	-5.96	0.038
K	intron retention	*MYO18A*	chr19	-	3.94	1.93	-2.01	0.040
N	alternative 3’ splice site	*MYOT*	chr7	+	3.97	3.51	-0.46	0.031
N	alternative 3’ splice site	*MYOZ1*	chr28	-	89.37	90.85	1.48	0.008
N	alternative 5’ splice site	*MYOZ3*	chr7	+	2.42	4.35	1.93	0.039
K	alternative first exon	*MYOZ3*	chr7	+	52.63	43.48	-9.15	0.037
N	alternative 5’ splice site	*NACA*	chr11	-	12.5	9.09	-3.41	0.011
K	alternative last exon	*NAP1L1*	chr5	-	5	3	-2	0.003
K	alternative 3’ splice site	*NAP1L4*	chr29	+	3.33	2.31	-1.02	0.005

K	cassette exon	*NBR1*	chr19	+	95.83	100	4.17	0.022
N	cassette exon	*NDUFS4*	chr20	-	95.24	98.64	3.4	0.039
N	alternative 5’ splice site	*NFE2L1*	chr19	-	10.53	3.36	-7.17	0.010
K	cassette exon	*NFE2L1*	chr19	-	71.88	68.29	-3.59	0.003
N	alternative 3’ splice site	*NFE2L1*	chr19	-	16.18	6.67	-9.51	0.012
N	alternative 3’ splice site	*NFE2L1*	chr19	-	1.02	4.92	3.9	0.023
N	alternative 3’ splice site	*NFE2L1*	chr19	-	81.18	90.32	9.14	0.038
N	alternative 5’ splice site	*NFE2L1*	chr19	-	87.47	94.63	7.16	0.011
N	cassette exon	*NR4A1*	chr5	-	100	92.7	-7.3	0.050
K	cassette exon	*NRBP1*	chr11	-	3.02	1	-1.02	0.038
K	cassette exon	*NTPCR*	chr28	+	4.08	1	-3.08	0.046
N	alternative 3’ splice site	*OAZ2*	chr10	+	89.58	100	10.42	0.050
N	alternative 5’ splice site	*OBSCN*	chr7	-	100	92.7	-7.3	0.027
N	alternative 5’ splice site	*P2RX5*	chr19	-	68.58	75	6.42	0.034
K	cassette exon	*PCBP2*	chr5	-	51.85	56.73	4.88	0.008
K	cassette exon	*PCBP2*	chr5	-	38.48	46.92	8.44	0.024
N	cassette exon	*PDLIM5*	chr6	-	96.37	97.84	1.47	0.005
N	cassette exon	*PDLIM5*	chr6	-	93.03	96.15	3.12	0.009
N	cassette exon	*PDLIM5*	chr6	-	1.7	1.05	-0.65	0.024
N	cassette exon	*PEBP4*	chr8	-	90.74	95.46	4.72	0.001
N	cassette exon	*PEBP4*	chr8	-	83.33	85.66	2.33	0.024
N	alternative 3’ splice site	*PERM1*	chr16	+	12.09	6.25	-5.84	0.007
N	alternative 3’ splice site	*PFKFB4*	chr22	+	93.62	95.61	1.98	0.046
N	alternative 3’ splice site	*PIP4K2B*	chr19	-	100	92.7	-7.3	0.026
N	alternative first exon	*PLXNB2*	chr5	-	95.24	96.3	1.06	0.048
N	alternative 3’ splice site	*POLR1D*	chr12	-	8.89	6.25	-2.64	0.038
K	cassette exon	*POLR2E*	chr2	+	98.15	100	1.85	0.027
N	coordinate cassette exon	*POLR2E*	chr7	-	97.62	100	2.38	0.040

N	alternative 3’ splice site	*PPFIA4*	chr16	+	89	92.73	3.73	0.004
K	cassette exon	*PPFIBP1*	chr5	-	77.46	63.33	-14.13	0.022
N	alternative 5’ splice site	*PPP2R2A*	chr8	+	4.55	2	-2.55	0.006
N	cassette exon	*PSMD1*	chr12	-	99.09	100	0.91	0.008
K	cassette exon	*PSMD14*	chr2	-	5.41	2.9	-2.52	0.017
N	alternative first exon	*PTP4A3*	chr14	-	8.72	4	-4.72	0.013
N	alternative first exon	*PTP4A3*	chr14	-	83.76	90.48	6.72	0.024
N	cassette exon	*RAB18*	chr13	-	100	100	0	0.042
K	alternative 3’ splice site	*RAD23A*	chr7	-	23.65	26.17	2.52	0.045
N	alternative 5’ splice site	*RAD9A*	chr29	-	4.76	3.39	-1.37	0.047
N	alternative 5’ splice site	*RAMP1*	chr3	+	96.77	100	3.23	0.042
N	alternative 3’ splice site	*RAPGEF1*	chr11	-	95.24	100	4.76	0.008
N	alternative 3’ splice site	*RAPGEF1*	chr11	-	4.95	1	-3.95	0.013
K	alternative 5’ splice site	*RB1CC1*	chr14	-	6.65	2	-4.65	0.035
N	alternative 3’ splice site	*RBL2*	chr18	+	87.5	100	12.5	0.000
N	alternative 3’ splice site	*RBM24*	chr23	-	3.42	2	-1.42	0.018
N	alternative 3’ splice site	*RBMX*	chrX	-	94.89	100	5.11	0.049
N	coordinate cassette exon	*RCHY1*	chr6	-	100	96	-4	0.022
N	alternative 5’ splice site	*RFNG*	chr19	+	7.02	9.52	2.5	0.034
N	alternative 5’ splice site	*RNASE4*	chr10	-	97.37	100	2.63	0.010
N	alternative 5’ splice site	*RNF7*	chr1	-	4.55	2	-2.55	0.017
N	alternative 5’ splice site	*RNF7*	chr1	-	9.12	3	-7.12	0.042
N	alternative 3’ splice site	*RPL3L*	chr25	-	94.91	96.75	1.84	0.043
N	alternative 5’ splice site	*RPLP1*	chr10	+	95.13	97.67	2.54	0.022
N	alternative 5’ splice site	*RPLP1*	chr10	+	4.87	2.33	-2.54	0.022
N	alternative 5’ splice site	*RPS26*	chr5	-	96.7	97.85	1.15	0.012
N	cassette exon	*RRAGD*	chr9	+	88.24	93.88	5.64	0.041
N	coordinate cassette exon	*RRAGD*	chr9	+	93.1	97.03	3.93	0.027

N	cassette exon	*RYK*	chr1	+	100	100	0	0.027
N	cassette exon	*SAR1A*	chr28	-	21.32	11.23	-10.09	0.014
N	alternative 3’ splice site	*SESN1*	chr9	+	2.33	1	-1.33	0.010
N	alternative 3’ splice site	*SFT2D1*	chr13	+	15.85	19.59	3.74	0.031
N	alternative 5’ splice site	*SFT2D1*	chr9	-	4.7	2	-2.7	0.042
N	alternative 3’ splice site	*SLC16A1*	chr3	+	94.67	100	5.33	0.024
K	alternative first exon	*SLC29A1*	chr23	+	99.09	100	0.91	0.009
N	alternative 3’ splice site	*SLC29A2*	chr29	-	4.54	2	-2.54	0.028
N	cassette exon	*SLC38A2*	chr5	+	100	100	0	0.047
K	cassette exon	*SMYD1*	chr11	-	51.43	60.87	9.44	0.013
K	cassette exon	*SORBS1*	chr26	-	97.47	100	2.53	0.047
K	alternative 5’ splice site	*SPEG*	chr2	+	95.45	97	1.55	0.037
K	cassette exon	*SRRM2*	chr25	-	47.87	33.52	-14.35	0.048
N	cassette exon	*ST3GAL3*	chr3	-	98.53	100	1.47	0.007
K	alternative 3’ splice site	*ST6GALN*	chr11	-	6.22	4	-2.22	0.005
N	coordinate cassette exon	*STAC3*	chr5	+	97.13	94.62	-2.51	0.038
K	alternative 3’ splice site	*STK11*	chr7	-	95.92	98.73	2.81	0.011
N	cassette exon	*STRN3*	chr21	-	96	100	-4	0.046
K	alternative last exon	*STX4*	chr25	+	96.77	95	-1.77	0.049
K	cassette exon	*SYNPO2L*	chr29	+	83.97	75	-8.97	0.020
N	alternative 5’ splice site	*TACC2*	chr26	+	55.83	50	-5.83	0.036
N	cassette exon	*TAX1BP1*	chr4	-	96.3	100	3.7	0.004
K	coordinate cassette exon	*TAX1BP1*	chr4	-	96.43	100	3.57	0.007
K	cassette exon	*TFDP2*	chr1	+	24.72	20	-4.72	0.009
K	cassette exon	*THRAP3*	chr5	+	17.31	13.58	-3.73	0.011
N	alternative 3’ splice site	*THYN1*	chr15	-	4.92	3	-1.92	0.009
K	alternative first exon	*THYN1*	chr15	-	5.48	3	-2.48	0.015
N	alternative 3’ splice site	*TIAL1*	chr26	-	81.97	85.44	3.47	0.029

N	alternative 3’ splice site	*TIAL1*	chr26	-	17.91	14.11	-3.8	0.035
N	cassette exon	*TMEM189*	chr13	-	33.52	47.87	14.35	0.046
N	cassette exon	*TMEM259*	chr7	-	100	96.66	-3.34	0.041
N	coordinate cassette exon	*TNS1*	chr2	-	86.41	97.06	10.66	0.006
K	alternative 5’ splice site	*TOB2*	chr5	-	99.09	100	0.91	0.049
K	alternative 3’ splice site	*TRIP12*	chr2	-	4.04	2	-2.04	0.009
K	cassette exon	*TWF2*	chr22	+	98.1	97.63	-0.47	0.035
N	alternative 3’ splice site	*UBAC2*	chr12	+	4.13	1	-3.13	0.023
K	cassette exon	*UBE2D1*	chr26	-	96.55	100	3.45	0.006
K	cassette exon	*UBE3A*	chr21	-	98.39	100	1.61	0.006
K	alternative 3’ splice site	*UBE3C*	chr4	+	67.06	63.64	-3.42	0.019
N	alternative 5’ splice site	*UBE3C*	chr6	+	78.76	84.31	5.55	0.006
N	alternative 5’ splice site	*UBE3C*	chr6	+	21.54	10	-11.54	0.046
N	alternative 5’ splice site	*UBL4A*	chrX	-	25	23.61	-1.39	0.046
K	alternative 3’ splice site	*UCHL5*	chr16	+	22.81	20	-2.81	0.025
N	alternative 3’ splice site	*ULK1*	chr17	-	47.87	33.52	-14.35	0.033
K	cassette exon	*UQCC*	chr13	-	100	96	-4	0.045
K	alternative first exon	*USP2*	chr15	-	82.35	90	7.65	0.016
K	alternative 3’ splice site	*USP28*	chr15	-	23.73	16.03	-7.7	0.035
N	alternative 5’ splice site	*USP47*	chr15	-	97.37	100	2.63	0.013
N	alternative 3’ splice site	*UXS1*	chr11	+	99.09	100	0.91	0.019
N	cassette exon	*WIPI1*	chr19	+	94.19	100	5.81	0.035
N	alternative 3’ splice site	*XIRP1*	chr22	-	82.84	86.32	3.48	0.050
K	cassette exon	*XIRP2*	chr2	-	87.75	94.74	6.99	0.020
N	cassette exon	*YIF1A*	chr29	-	96.49	90	-6.49	0.042
K	cassette exon	*ZMYND11*	chr13	-	51.15	38.89	-12.26	0.002

Table S4. Biological process Gene ontology (GO) terms and pathways enriched for genes with differentially expressed alternatively spliced transcripts in ribeye muscle of Nelore bulls selected to be divergent for intramuscular fat content

	**Terms**	**Genes**	**p-*value***
Biological Process	GO:0042787~protein ubiquitination involved in ubiquitin-dependent protein catabolic process	*ARIH2, RNF7, UBE3A, UBE3C, ANAPC11, RCHY1, TRIP12*	0.003
	GO:0016579~protein deubiquitination	*USP28, COPS5, USP2, USP47, UCHL5*	0.004
	GO:0030239~myofibril assembly	*MYOZ1, MYOZ3, LMOD3*	0.007
	GO:0035914~skeletal muscle cell differentiation	*MEF2C, MEF2D, NR4A1, SMYD1*	0.019
	GO:0006366~transcription from RNA polymerase II promoter	*MEF2C, POLR2E, HNRNPK, NR4A1, NFE2L1, RBMX*	0.024
	GO:0043484~regulation of RNA splicing	*MBNL1, HNRNPH1, AHNAK*	0.031
	GO:0001958~endochondral ossification	*MEF2C, MEF2D, INPPL1*	0.034
	GO:0006511~ubiquitin-dependent protein catabolic process	*USP28, USP2, USP47, UCHL5, UBE2D1*	0.037
	GO:0023051~regulation of signaling	*LMO7, MVP*	0.038
	GO:0007274~neuromuscular synaptic transmission	*KIF1B, MYLK2, STAC3*	0.042
	GO:0006099~tricarboxylic acid cycle	*CS, IDH3B, FH*	0.042
	GO:0051262~protein tetramerization	*UXS1, CUTC, FH*	0.058
	GO:2001237~negative regulation of extrinsic apoptotic signaling pathway	*ZMYND11, CTTN, RB1CC1*	0.062
	GO:0034644~cellular response to UV	*KDM1A, USP28, USP47*	0.062
	GO:1904948~midbrain dopaminergic neuron differentiation	*RYK, CTNNB1*	0.062
	GO:0045944~positive regulation of transcription from RNA polymerase II promoter	*MEF2C, COPS5, UBE3A, STRN3, NR4A1, RBMX, CTNNB1, KDM1A, HSF1, THRAP3, ARMCX3, NFE2L1, AKIRIN2*	0.065
	GO:0023052~signaling	*DLGAP4, TOB2*	0.074
	GO:0070125~mitochondrial translational elongation	*MRPS18A, MRPS25, TSFM, MRPL33*	0.079
	GO:1904262~negative regulation of TORC1 signaling	*STK11, SESN1*	0.085
	GO:0055008~cardiac muscle tissue morphogenesis	*XIRP2, MYLK2*	0.097
	GO:0048643~positive regulation of skeletal muscle tissue development	*MEF2C, CTNNB1*	0.097
Pathways	bta04120:Ubiquitin mediated proteolysis	*RNF7, UBE3A, UBE3C, ANAPC11, RCHY1, UBE2D1, TRIP12*	0.003

bta04150:mTOR signaling pathway	*EIF4B*, *ULK1*, *STK11*, *RRAGD*	0.025
bta00020:Citrate cycle (TCA cycle)	*CS*, *IDH3B*, *FH*	0.040
bta03040:Spliceosome	*HNRNPK*, *DDX39B*, *RBMX*, *BUD31*, *HNRNPA1*	0.049
bta05169:Epstein-Barr virus infection	*PSMD14*, *POLR2E*, *POLR1D*, *PSMD1*, *CD58*, *MAP2K6*	0.054
bta03010:Ribosome	*RPS26*, *MRPS18A*, *RPLP1*, *RPL3L*, *MRPL33*	0.055
bta04151:PI3K-Akt signaling pathway	*EIF4B*, *HSP90AB1*, *GNB2*, *RBL2*, *STK11*, *COL6A2*, *NR4A1*, *PPP2R2A*	0.077
